# Hsa_circ_0000479 as a Novel Diagnostic Biomarker of Systemic Lupus Erythematosus

**DOI:** 10.3389/fimmu.2019.02281

**Published:** 2019-09-24

**Authors:** Gangqiang Guo, Huijing Wang, Lele Ye, Xinyu Shi, Kejing Yan, Kangmin Lin, Qunjia Huang, Baoqing Li, Qiaoai Lin, Lejiang Zhu, Xiangyang Xue, Huidi Zhang

**Affiliations:** ^1^Department of Microbiology and Immunology, School of Basic Medical Sciences, Institute of Molecular Virology and Immunology, Institute of Tropical Medicine, Wenzhou Medical University, Wenzhou, China; ^2^Kidney Disease Center, First Affiliated Hospital, College of Medicine, Zhejiang University, Hangzhou, China; ^3^Department of Rheumatology, Renji Hospital, Shanghai Jiaotong University School of Medicine, Shanghai, China; ^4^Department of Laboratory Medicine, Second Affiliated Hospital and Yuying Children's Hospital, Wenzhou Medical University, Wenzhou, China; ^5^Department of Nephrology, Wenzhou Central Hospital, Wenzhou, China; ^6^Department of Nephrology, First Affiliated Hospital, Wenzhou Medical University, Wenzhou, China

**Keywords:** RNA-sequencing, systemic lupus erythematosus, circular RNAs (circRNAs), hsa_circ_0000479, biomarker

## Abstract

**Background:** Accumulating evidence suggests that differentially expressed non-coding circular RNAs (circRNAs) play critical roles in the progress of autoimmune diseases. However, the role of circRNAs in systemic lupus erythematosus (SLE) remains unclear.

**Methods:** We initially used next-generation sequencing (NGS) to comprehensively analyze circRNA expression profiles in peripheral blood mononuclear cells (PBMCs) from 10 SLE patients, stratified by their disease activity characteristics (stable or active SLE), and 10 healthy controls (HCs). Candidate circRNAs identified were first validated by quantitative reverse-transcription (qRT)-PCR in PBMC samples from a training-phase cohort of five SLE patients and five HCs. The significantly dysregulated circRNAs were then confirmed by qRT-PCR in a validation cohort of 23 SLE patients and 21 HCs, and in an external validation cohort with 64 SLE patients, 58 HCs, and 50 patients with rheumatoid arthritis (RA). In addition, we conducted bioinformatics analysis and western blotting investigating the relationships between the candidate circRNAs and SLE progression.

**Results:** Multilayer integrative analysis of circRNA regulation showed that 84 circRNAs were upregulated and 30 were downregulated in patients with SLE compared with HCs. We then analyzed the intersection of these differentially expressed circRNAs in an SLE-stable cohort, an SLE-active cohort, and HCs. This enabled us to narrow down dysregulated circRNAs to 15 upregulated circRNAs. Only hsa_circ_0000479 was significantly upregulated in PBMCs of patients with SLE compared with HCs (*P* < 0.05). Furthermore, the diagnostic potential of hsa_circ_0000479 expression to distinguish SLE patients from HCs and RA patients was also significantly increased in the validation-phase and external-validation-phase cohorts (*P* < 0.05). When distinguishing SLE patients from HCs, the diagnostic specificities of hsa_circ_0000479 were 0.619 and 1.0 in two validation cohorts, respectively (AUCs = 0.731 and 0.730, respectively). It was also significantly increased in either stable SLE patients or active SLE patients compared with HCs in these two cohorts (*P* < 0.05). We also used bioinformatics analysis to show that hsa_circ_0000479 regulates SLE progression by modulating metabolic pathways and the Wnt signaling pathway. Western blotting revealed that the expression of Wnt-16 protein was significantly decreased in SLE.

**Conclusion:** Our results suggest that hsa_circ_0000479 has potential as a novel biomarker for the diagnosis of SLE.

## Introduction

Systemic lupus erythematosus (SLE) is a chronic and complex autoimmune disease, mediated by pathogenic autoantibodies, with severe clinical manifestations such as nephritis and multisystem organ failure (1). The global prevalence rates of SLE vary from ~20 to 150 per 100,000 people (2), with a male to female ratio of 1:9 (3). Various genetic and environmental factors contribute to the development of SLE. As the etiology and pathogenesis underlying its development remain unclear, SLE is still associated with high morbidity and mortality. Although there is a growing number of advancements focused on identification of biomarkers to prevent disease progress and improve clinical outcomes, prognosis remains poor for some patients (4). A better understanding of biomarkers associated with SLE will not only increase knowledge of disease pathogenesis, but inform prognostication (5, 6). It could also potentially help in the development of novel targeted therapies.

Recent studies have found close associations between SLE pathogenesis and aberrantly expressed non-coding RNAs (ncRNAs) including microRNAs and circular RNAs (circRNAs). These works have suggested the potential of these RNAs as biomarkers for the diagnosis and progress of the disease. Evidence has indicated a key role in the development of SLE for several ncRNAs (7–9). A class of ncRNAs, circRNAs are formed of loops covalently closed by linking of their 5′ and 3′ ends (10, 11). Due to this structure, they have a high level of stability (12). This increases their effectiveness as microRNA sponges to regulate the expression of parental genes via the competing endogenous RNA (ceRNA) network (13).

Recently, one study used microarray detection to show different expression of circular RNAs in plasma samples from SLE and healthy controls (HCs), and validated these findings in peripheral blood mononuclear cells (PBMCs) (8). However, despite knowledge of many molecular mechanisms of ncRNA function in SLE, and the identification of several potential circRNA biomarkers, the role of circRNA in PBMCs from different disease stratifications in SLE has not been fully elucidated. Different patients with different disease activity may have different treatment responses and prognosis (14). Disease stratifications in SLE could improve their therapy and achieve greater efficacy (15).

The purpose of this study was to explore the hypotheses that differentially expressed circRNAs may be involved in the progression of SLE, and that they represent promising novel biomarkers. We firstly evaluated the expression profiles of circRNAs in PBMCs from Chinese SLE patients and normal individuals by next-generation sequencing (NGS). Secondly, intersecting dysregulated circRNAs from a normal-stable differentiated set and a normal-active differentiated set to narrow candidate circRNAs, we constructed a predictive model for the circRNA-miRNA regulatory network. Finally, we validated the differentially expressed cicrRNAs in SLE patients compared with HCs and patients with rheumatoid arthritis (RA).

## Materials and Methods

### Subjects and Study Design

This study included 256 participants and four phases, as detailed in Supplementary Table 1. In the discovery phase, we first conducted NGS to analyze circRNA expression profiles in PBMCs from 10 SLE patients, stratified by their disease activity characteristics, and 10 HCs. In the training phase, the expression of candidate circRNAs in PBMCs were explored by qRT-PCR in 10 samples (five HCs and five patients with SLE). These 10 samples were used to further screen the candidate circRNAs with the following criteria: (1) an expression trend in the same direction as in the RNA-seq analysis results; (2) a statistically significant expression level difference between SLE patients and HCs. The diagnostic parameters of hsa_circ_0000479 were calculated in the training phase, and further assessed in 44 subjects (eight patients with SLE at a stable stage, 15 patients with SLE at an active stage, 21 HCs) in the validation phase. In the external validation phase, the diagnostic role of hsa_circ_0000479 was independently evaluated in an additional 172 subjects (28 patients with SLE at a stable stage, 32 patients with SLE at an active stage, four patients we were unable to classify due to lack of necessary clinical information, 58 HCs, and 50 patients with RA). In addition, PBMCs from five HCs and five SLE patients were selected randomly to validate the Wnt-16 protein expression of hsa_circ_0000479's potential target gene. The clinical characteristics of these subjects are shown in Supplementary Table 1, and the screening flow chart is shown in Supplementary Figure 1.

Thirty-eight SLE patients admitted to the Department of Rheumatology, First Affiliated Hospital of Wenzhou Medical University, between January 2018 and July 2019, were enrolled in this study. These patients were assigned randomly to the discovery-phase, training-phase, or validation-phase cohorts. An additional 64 SLE patients and 50 RA patients were enrolled from June 2018 to June of 2019 from the Department of Rheumatology, Renji Hospital of Shanghai Jiao Tong University. These patients were allocated to the external validation-phase cohort. All SLE patients fulfilled the American College of Rheumatology (ACR) 1997 (16) and all RA patients fulfilled the ACR 2010 criteria (17). As the standard of classification, disease activity was assessed according to the systemic lupus erythematosus disease activity index (SLEDAI) (18) at the time of blood collection. SLE patients with SLEDAI ≥ 5 were allocated to the active-disease cohort, and those with SLEDAI < 5 were allocated to the stable-disease cohort. Ninety-nine age- and sex-matched HCs without arthralgia, heart failure, renal failure, or autoimmune disease, and free from other inflammatory conditions, were recruited from Department of Laboratory Medicine, Second Affiliated Hospital & Yuying Children's Hospital, Wenzhou Medical University. The research protocol was approved by the Medical Ethical Committees of the First/Second Affiliated Hospital of Wenzhou Medical University and Renji Hospital, Shanghai Jiaotong University School of Medicine. All subjects who participated in this research provided written informed consent.

### PBMC and RNA Isolation

PBMCs were isolated from SLE patients, HCs and RA patients using human peripheral blood lymphocyte separation medium (Tianjin Hao Yang Biological Manufacture, Tianjin, China) within 4 h of collection of the samples. Total RNA was extracted from each sample using TriZol Reagent (Invitrogen Life Technologies®, Grand Island, NY, USA). The isolated RNAs were digested by Dnase I (Invitrogen™, Waltham, MA, USA) to remove residual DNA, and were then collected in 25 μL of DNase/RNase-free water. The concentration of RNA was quantified and qualified using a NanoDrop instrument (Thermo Fisher Scientific Inc., Waltham, MA, USA) and 1% agarose gel electrophoresis. Isolated RNA was stored at −80°C for use. The samples from the discovery phase cohort were used for NGS analysis. Samples from the training-phase, validation-phase, and external-validation-phase cohorts were used for validation by qRT-PCR.

### NGS Profiling

Total RNA was extracted from each sample using a TRIzol Reagent (Invitrogen)/RNeasy Mini Kit (Qiagen). Total RNA was quantified and qualified using an Agilent 2,100 Bioanalyzer (Agilent Technologies, Palo Alto, CA, USA), NanoDrop (Thermo Fisher Scientific Inc.) and 1% agarose gel. One microgram of total RNA with RIN value above seven was used for subsequent library preparation. NGS libraries were prepared according to the manufacturer's protocol (NEBNext® Ultra™ Directional RNA Library Prep Kit for Illumina®). The rRNA was depleted from total RNA using a Ribo-Zero™ rRNA removal Kit (Human/Mouse/Rat)/(Yeast) /(Bacteria) (Illumina, San Diego, CA, USA). The rRNA-depleted RNA was then fragmented and reverse-transcribed. Libraries with different indices were multiplexed and loaded on an Illumina HiSeq instrument according to the manufacturer's instructions (Illumina, San Diego, CA, USA). Sequencing was carried out using a 2 × 150 paired-end (PE) configuration; image analysis and base calling were conducted using HiSeq Control Software (HCS) + OLB + GAPipeline-1.6 (Illumina) on a HiSeq instrument.

For circRNA identification, we used BWA(0.7.12-r1039) to align clean data to the reference genome (19). We used the software CIRI (v2.0) to identify circRNAs from the alignment files (^*^.sam). The junction reads at the back-splicing loci of circRNA are used to calculate its expression. Spliced reads per billion mapping (SRPBM) was used to normalize the reads. For differential expression analysis, we used the DESeq Bioconductor package, a model based on the negative binomial distribution. After adjustment by Benjamini and Hochberg's method for controlling the false discovery rate, the threshold values for differential expression were set at padj < 0.05, and fold change > 2.

For mRNA identification, Hisat2 (v2.0.1) was firstly used to index the reference genome sequence. Clean data were then aligned to the reference genome using Hisat2 software (v2.0.1). In this process, transcripts in fasta format were initially converted from known gff annotation files and indexed properly. Then, with the file as a reference gene file, HTSeq (v0.6.1) estimated gene and isoform expression levels from the pair-end clean data. Differential expression analysis was performed using the DESeq Bioconductor package, a model based on the negative binomial distribution. After adjustment by Benjamini and Hochberg's approach for controlling the false discovery rate, thresholds for differentially expressed genes were set at padj < 0.05, and fold change > 1.5. In this study, we do not show the full data on differentially expressed genes in PBMCs between SLE patients and HCs.

### Construction of circRNA-miRNA Co-expression Network

The human mature miRNA sequence was downloaded from the miRNA database (http://www.mirbase.org). Based on the predicted circRNA site information from all the sequenced samples, circRNA sequence was extracted, and the miRNA binding sites of circRNAs were predicted with the software miRanda-3.3a. In order to investigate the functions of the candidate disease stage-related circRNAs, their potential miRNA binding sites were predicted. Based on candidate significantly differentially expressed circRNAs and mRNAs, and their common predicted miRNA binding sites, a co-expression network of circRNA-miRNA or circRNA-miRNA-mRNA was established using Cytoscape 3.7.0.

### qRT-PCR Validation of Candidate circRNAs

From the 15 candidate circRNAs identified in our comprehensive analysis, 10 representative circRNAs were selected and validated by qRT-PCR in the training-phase cohort. The hsa_circ_0000479 expression levels in the PBMCs from 44 members of the validation-phase cohort and 172 members of the external validation cohort were detected by qRT-PCR using a TUREscript 1st stand cDNA SYNTHESIS Kit (Aidlab, Beijing, China). All qRT-PCR reactions were carried out on a -qTOWER 2.2 Real-Time PCR system (Analytik Jena, Germany). For each reaction, 1 μL of diluted cDNA was mixed with 5 μL of 1 × SYBR Green Reaction Mix (DBI, Ludwigshafen, Germany). A final volume of 10 μL was achieved by the addition of 200 nM of forward and reverse primers. The conditions for PCR amplification were as follows: 95°C for 3 min, followed by 44 cycles of 95°C for 10 s and 58°C for 30 s. The fluorescence signal was measured once every 1°C. The specificity of the primer amplicons was tested by melting curve analysis. All samples were tested in triplicate. The data were analyzed using the comparative threshold cycle (Ct) method. GAPDH was used as a control, and the relative quantification of circRNAs in PBMCs was calculated using the following equation: amount of target = 2^−Δ*Ct*^, where ΔCt = Ct _PBMCcirciRNAs_ – Ct _GAPDH_. The gene-specific primers used are listed in Supplementary Table 2.

### GO and KEGG Pathway Analysis

The functions of the target genes of 12 differentially expressed circRNAs were investigated using gene ontology (GO, http://geneontology.org/) annotations and Kyoto encyclopedia of genes and genome (KEGG, http://www.kegg.jp/) enrichment analysis. Hierarchical clustering of the differentially expressed genes (DEGs) according to the biological process (BP), cellular component (CC), and molecular function (MF) categories was performed by GO analysis to elucidate genetic regulatory networks. Pathway analysis using graphical diagrams was performed to explore DEG pathways using the KEGG database. Moreover, to further investigate the functions of candidate hsa_circ_0000479, GO analysis using the PANTHER database (http://www.pantherdb.org) was conducted. Significance was determined from *P*-value (*P* < 0.05) and *Q*-values. To further investigate the biological functions and most revelant networks of the function of candidate circRNA target genes in SLE, Thomson Reuters database (https://portal.genego.com) (20, 21) was used.

### Western Blotting

PBMCs from five healthy controls and five SLE patients were lysed using protein lysis buffer (Beyotime Institute of Biotechnology, Beijing, China) supplemented with protease inhibitor cocktail (Pierce, Rockford, IL, USA) at 4°C for 20 min. Protein samples were separated using 10% sodium dodecyl sulfate (SDS)-polyacrylamide gel electrophoresis and then electrophoretically transferred to polyvinylidene difluoride membranes (Millipore,Billerica, MA, USA). Anti-Wnt 16 antibody (ab109437, abcam, Cambridge, UK) was diluted with primary antibody dilution buffer (Beyotime Institute of Biotechnology) to 1:1,000, and anti-GAPDH antibody (GOOD HERE, Hangzhou, China) was also diluted 1:1,000. The membranes were then washed with TBST buffer five times for 5 min each and incubated with horseradish peroxidase (HRP)-conjugated goat antirabbit IgG secondary antibody (1:5,000 dilution) (MULTI SCIENCES) for 1.5 h at 37°C. Bands were detected using enhanced chemiluminescence and visualized with a Gel Doc 2,000 (BioRad, Hercules, CA, USA).

### Statistical Analysis

Statistical analysis was performed with SPSS 22.0 software (SPSS, Inc., Chicago, IL, USA). The non-parametric Mann-Whitney *U* test or independent-sample *T* test was performed to analyze correlations between hsa_circ_0000479 expression and clinical features of SLE. These data are presented as mean ± standard deviation. The non-parametric Mann-Whitney *U* test was used to double-validate *hsa_circ_0000479* as an SLE diagnosis marker in two additional cohorts and the data are presented as 50% quantile (25% quantile, 75% quantile). A two-sided *p* < 0.05 was considered to represent a statistically significant difference. Receiver operating characteristic (ROC) curve analysis, plotting the true positive rate (sensitivity) vs. the false positive rate (1 – specificity), at various threshold settings, was performed for PBMC circRNAs, and the areas under curve (AUC) were calculated using SPSS 22.0 software (SPSS, Inc., Chicago, IL, USA). The maximum of the sum of the true positive rate and false positive rate was calculated, and a cutoff value with higher specificity was selected. Expression graphs and ΔCt values were analyzed using GraphPad Prism version 5.04 software (GraphPad Software, La Jolla, CA, USA).

## Results

### Fifteen circRNAs Were Associated With the Disease Activity of SLE Patients

**Figure 1:**
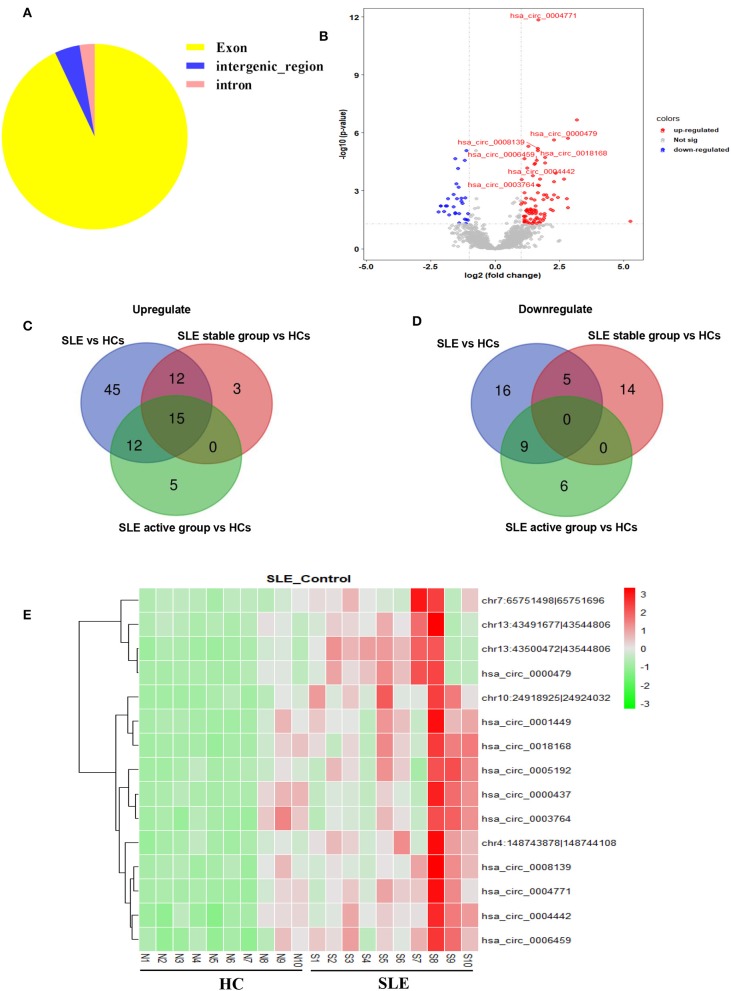
Sequencing data on differential circRNA expression profiles in PBMCs from SLE patients and HCs in the discovery-phase cohort. **(A)** The percentage of significantly differentially expressed circRNAs arising from different genomic regions (exon, intron, and intergenic regions). **(B)** Volcano plot of differentially expressed circRNAs. The blue spots indicate significantly downregulated circRNAs, and the red spots indicate significantly upregulated circRNAs. **(C,D)** The overlapping significantly differentially expressed circRNAs in PBMCs of SLE patients vs. HCs. There were 84 significantly upregulated **(C)** and 30 downregulated **(D)** circRNAs in PBMCs of SLE patients vs. HCs (blue area). There were 30 significantly upregulated **(C)** and 19 downregulated **(D)** circRNAs in PBMCs of the SLE-stable group vs. HCs (red area). There were 32 significantly upregulated **(C)** and 15 downregulated **(D)** circRNAs in PBMCs of the SLE-active group vs. HCs (green area). Integrating these three comparisons, we found 15 overlapping significantly upregulated circRNAs in PBMCs of SLE patients vs. HCs. These 15 significantly altered circRNAs are detailed in Supplementary Table 4. **(E)** Hierarchical clustering of the differentially expressed circRNAs. Red represents relatively highly expressed circRNAs, and green represents relatively lowly expressed circRNAs.

We first determined the expression profile of circRNAs in PBMC samples from 10 SLE patients and 10 HCs (clinical information of patients is detailed in Supplementary Table 1). Using fold-change filtering (fold change > 2) and padj < 0.05, the sequencing results identified 114 significantly differentially expressed circRNAs from the twenty samples (Supplementary Table 3). And 106 (~93%) of these circRNAs were derived from exons (Figure 1A). Compared with HCs, 84 and 30 circRNAs were upregulated and downregulated in SLE patients, respectively. A volcano plot showed the variance among DEGs (Figure 1B).

Second, we used cluster screening to analyze the effects of disease activity against the background of healthy control samples. This enabled identification of circRNAs associated with SLE disease activity. Our disease-stable cohort consisted of ten SLE patients, five of whom had active disease. Calculating the intersection of differentially expressed circRNAs from comparisons of the healthy-control, disease-stable, and disease-active cohorts, we were able to narrow candidate circRNAs down to 15 (Supplementary Table 4). A Venn diagram of this clustering shows that these consisted of 15 significantly upregulated circRNAs and zero downregulated circRNAs overlapping between the foregoing comparisons (Figures 1C,D). The 15 candidate differentially expressed circRNAs were visualized using heat mapping (Figure 1E).

### Network Construction of circRNA-miRNA Interactions, and GO and KEGG Pathway Analysis of the 15 Candidate circRNA

**Figure 2:**
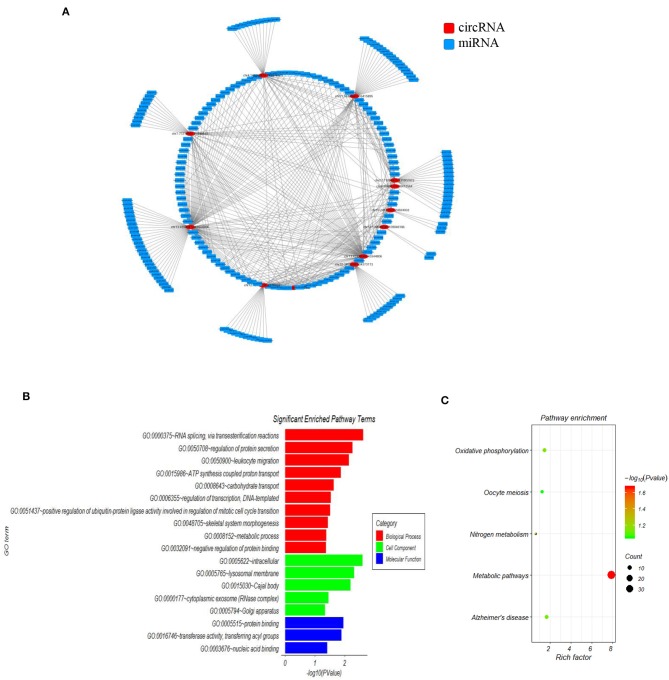
The mapping network of circRNA-miRNA interactions in SLE. **(A)** The network map includes the 12 surviving candidates from the 15 significantly altered circRNAs (represented as red nodes) in the analysis for circRNA-miRNA network prediction. The other three differentially expressed circRNAs did not show reliable results in this interaction analysis. The blue nodes around the red node were the predicted miRNAs that interacted with the related circRNAs. **(B)** Gene ontology (GO) analysis for 12 circRNA-interacting miRNAs and their target genes showing significantly enriched pathways. Red indicates biological process (BP), green indicates molecular function (MF), and blue indicates cellular component (CC). **(C)** KEGG Pathway analysis for 12 circRNA-interacting miRNAs and their target genes showing significantly enriched signaling pathways. The Y-axis indicates pathway name, and the X-axis indicates the richness factor. The size of the spots represents the number of enriched differential target genes, and change of color from green to red represents the Q-value.

Accumulating evidence has shown that circRNAs regulate gene expression by serving as miRNA sponges in some conditions. In order to investigate the functions of the 15 SLE disease stage-related circRNAs, their potential miRNA binding sites were predicted and complex circRNA-miRNA network for the 15 candidate circRNAs was constructed. As shown in Figure 2A, except for three circRNAs without ceRNA interaction, all differentially expressed circRNAs and predicted miRNAs interacted in the network. Within the network, between three and 115 miRNAs could target a single circRNA. Based on the circRNA-miRNA network, we confirmed all interacting miRNAs for each SLE-related circRNA through specific base-pair alignment.

Additionally, we investigated the functions of identified target genes using GO and KEGG pathway analyses. GO analysis showed that the target genes associated with candidate circRNAs were involved in some essential biological functions. In the BP category, enriched terms included RNA splicing, regulation of protein secretion, ATP synthesis-coupled proton transport, and metabolic process. In the CC category, enriched terms included intracellular, lysosomal membrane, and cytoplasmic exosome. In the MF category, enriched terms included protein binding, transferase activity, and nucleic acid binding (Figure 2B). KEGG pathway analysis showed that the candidate circRNAs participated in several key pathways, including metabolic pathways, oxidative phosphorylation, and nitrogen metabolism (Figure 2C). These functional analyses therefore identified relevant metabolic pathways that are important in the development of autoimmune disease.

### hsa_circ_0000479 Acts as a Novel Potential Diagnostic Biomarker for SLE

**Figure 3:**
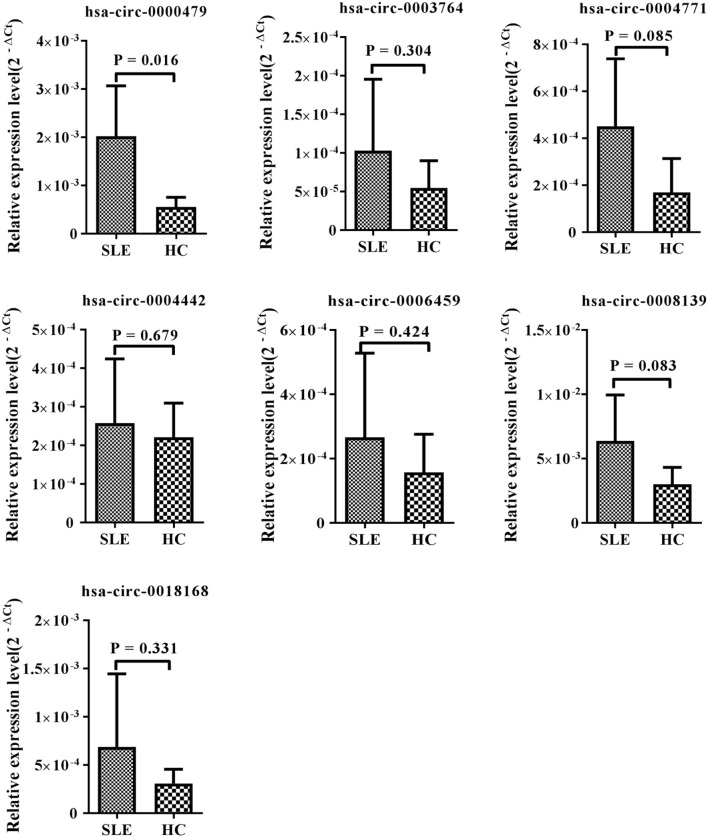
Expression of candidate circRNAs in PBMCs of SLE patients and healthy controls. The expression trends of seven circRNAs was consistent with the NGS profile results. qRT-PCR was conducted on RNA samples from five SLE patients and five HCs. Data are presented as 2^−Δ*Ct*^ relative to GAPDH expression (mean ± standard deviation).

Among the 15 candidate circRNAs, based on predicted softwares, five had zero or multiple (≥2) annotated_circRNA_IDs (novel circRNAs) excluded from this study. The ten remaining significantly differentially expressed circRNAs (hsa_circ_0001449, hsa_circ_0008139, hsa_circ_0004442, hsa_circ_0000437, hsa_circ_0000479, hsa_circ_0006459, hsa_circ_0003764, hsa_circ_0004771, hsa_circ_0005192, and hsa_circ_0018168) were chosen for validation in the training-phase cohort consisting of five SLE patients and five healthy controls. Specific primers targeting each circRNA were designed, and successfully amplified the candidate circRNAs, with the exception of hsa_circ_0005192, which could not be amplified and was therefore excluded from this research. The results of qRT-PCR, showed expression trends consistent with our sequencing results for seven circRNAs, whereas the other two circRNAs, showed an opposite trend to that detected by NGS. However, only the expression of hsa_circ_0000479 was significantly increased in SLE patients in the training-phase cohort (*P* < 0.05; Figures 1B, 3). This suggested that hsa_circ_0000479 might be a novel potential diagnostic biomarker for SLE.

**Figure 4:**
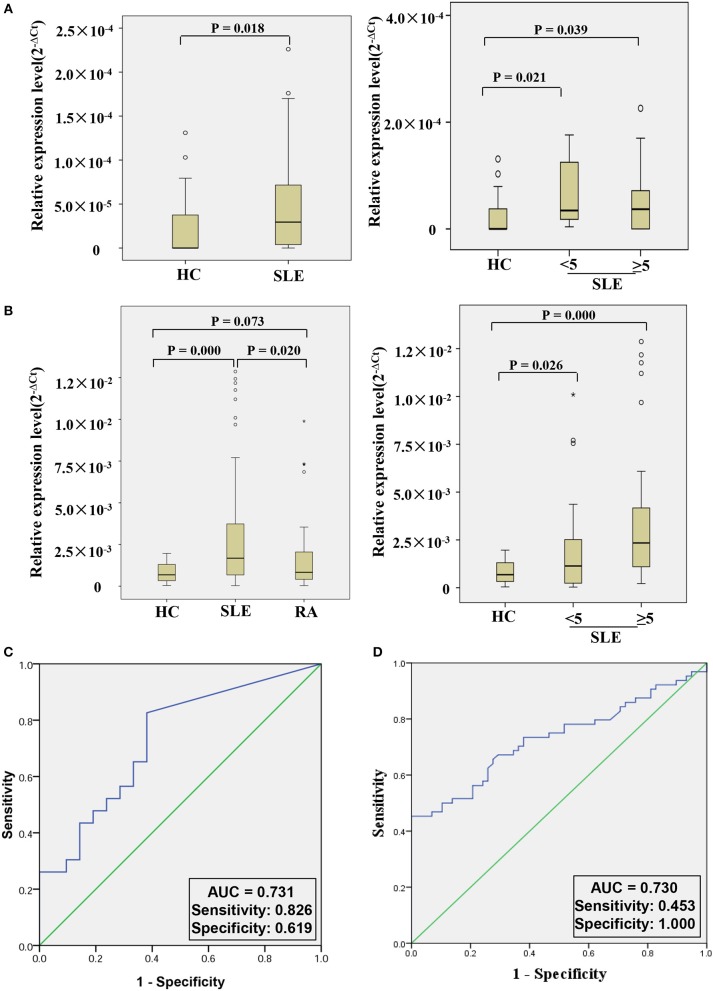
Double validation of hsa_circ_0000479 as an SLE diagnosis marker in two additional cohorts. **(A)** Expression of hsa_circ_0000479 in the validation-phase cohort with 23 SLE patients (including the active group and the stable group) and 21 HCs. **(B)** Expression of hsa_circ_0000479 in the external validation phase cohort with 64 SLE patients (including the active group and the stable group), 58 HCs, and 50 RA patients. Data are presented as a box plot. The “°” and “*,” respectively, indicate data that are more than 1.5-fold and 3-fold the quartile distance from the upper or lower bounds of the box. **(C,D)** Receiver operating characteristic (ROC) curves of hsa_circ_0000479 in the validation-phase and external validation-phase cohorts for SLE diagnosis.

In order to further explore whether hsa_circ_0000479 could be a useful biomarker, we tested it in a larger validation-phase cohort of 23 SLE patients and 21 HCs, and another larger external validation-phase cohort of 64 SLE patients, 58 HCs, and 50 RA patients using qRT-PCR. Compared with HCs, hsa_circ_0000479 expression in SLE-patients' PBMCs was significantly increased in both validation-phase and external validation-phase cohorts (*P* = 0.018 and 0.000, respectively; Figures 4A,B). Compared with RA patients, its expression in SLE-patients' PBMCs was significantly increased in the external validation-phase cohort (*P* = 0.020; Figure 4B). However, hsa_circ_0000479 expression in PBMCs showed no difference between RA patients and HCs. Moreover, in order to explore whether hsa_circ_0000479 could distinguish among different disease-stage SLE patients (stable or active stage) and HCs, its expression level was further analyzed according to disease stage. The results showed that hsa_circ_0000479 could significantly distinguish between stable- or active- SLE patients and HCs in both the validation-phase cohort (*P* = 0.021 and 0.039, respectively; Figures 4A,B) and the external validation-phase cohort (*P* = 0.026 and 0.000, respectively; Figures 4A,B).

We also used ROC curve analysis to further validate the potential utility of hsa_circ_0000479 as a diagnostic biomarker of SLE. When distinguishing SLE patients from healthy persons, AUCs for hsa_circ_0000479 were 0.731 and 0.730 in the validation-phase and external validation phase cohorts, respectively (Figures 4C,D). In these two validation cohorts, the diagnostic sensitivities of hsa_circ_0000479 were 0.826 and 0.453, respectively, and its specificities were 0.619 and 1.0, respectively (Figures 4C,D).

### Correlation Between hsa_circ_0000479 Expression and SLE Clinical Characteristics

The relationship between hsa_circ_0000479 expression levels in PBMCs and the clinical features of 64 SLE patients was further analyzed in the external validation phase cohort (Table 1). The results showed that high expression of hsa_circ_0000479 in SLE patients was significantly more relevant with low albumin level (*P* = 0.002), positive urine protein (*P* = 0.005), positive anticardiolipin antibody IgG (*P* = 0.028), low leukocytes (*P* = 0.043), low hemoglobin (*P* = 0.015), high total IgG (*P* = 0.021), and high ESR (*P* = 0.0002).

**Table 1:** Correlation of the expression of the hsa_circ_0000479 and clinical features of SLE.

**Clinical characteristics**		**N**	**hsa_circ_0000479**	***P*-value^#^**
Anti-ds-DNA antibody	Positive	27	0.0028 ± 0.0037	0.279
	Negative	31	0.0030 ± 0.0032	
Anti-Rib-P antibody	Positive	12	0.0031 ± 0.0033	0.443
	Negative	46	0.0029 ± 0.0035	
Anti-Smith-antibody	Positive	12	0.0034 ± 0.0032	0.249
	Negative	46	0.0028 ± 0.0035	
Anti-SSA antibody	Positive	33	0.0032 ± 0.0035	0.435
	Negative	25	0.0025 ± 0.0033	
Anti-SSB antibody	Positive	7	0.0031 ± 0.0037	0.591
	Negative	51	0.0029 ± 0.0034	
Direct Coomb's test	Positive	8	0.0046 ± 0.0049	0.291
	Negative	33	0.0021 ± 0.0027	
Indirect Coomb's test	Positive	4	0.0021 ± 0.0014	0.596
	Negative	37	0.0027 ± 0.0034	
β2-GP1	Positive	11	0.0014 ± 0.0021	0.242
	Negative	26	0.0019 ± 0.0020	
Anticardiolipin antibody IgG	Positive	21	0.0042 ± 0.0043	**0.028**
	Negative	31	0.0018 ± 0.0019	
Lupus anticoagulant	0.75 ~ 1.25	23	0.0034 ± 0.0036	0.115
	>1.25	13	0.0019 ± 0.0026	
Leukocytes	0 ~ 3.0	7	0.0053 ± 0.0048	**0.043**
	>3.0	53	0.0026 ± 0.0030	
Hemoglobin	<110	36	0.0034 ± 0.0035	**0.015**
	110 ~ 150	24	0.0021 ± 0.0030	
Platelet	100 ~ 350	44	0.0027 ± 0.0031	0.813
	<100	14	0.0030 ± 0.0037	
Albumin	<40	39	0.0034 ± 0.0035	**0.002**
	≥40	13	0.0012 ± 0.0021	
ALT	>75	5	0.0045 ± 0.0041	0.255
	0 ~ 75	55	0.0027 ± 0.0033	
AST	>35	13	0.0033 ± 0.0034	0.293
	0 ~ 35	41	0.0030 ± 0.0033	
C3^*^	<0.9	50	0.0028 ± 0.0033	0.214
	0.9 ~ 1.8	9	0.0038 ± 0.0038	
C4^*^	<0.1	24	0.0033 ± 0.0035	0.419
	0.1 ~ 0.4	33	0.0025 ± 0.0031	
Urine protein	Positive	28	0.0038 ± 0.0035	**0.005**
	Negative	26	0.0021 ± 0.0033	
Urine occult blood	Positive	16	0.0029 ± 0.0034	0.860
	Negative	39	0.0029 ± 0.0035	
Serum creatinine	>104	13	0.0038 ± 0.0041	0.280
	45 ~ 104	38	0.0026 ± 0.0034	
Total IgG	>16	18	0.0045 ± 0.0045	**0.021**
	≤ 16	37	0.0022 ± 0.0026	
Total IgM	>2.3	2	0.0029 ± 0.0006	0.421
	≤ 2.3	54	0.0029 ± 0.0035	
Total IgA	>4.0	6	0.0040 ± 0.0048	0.703
	≤ 4.0	49	0.0028 ± 0.0033	
CRP	0 ~ 8	46	0.0026 ± 0.0032	0.089
	>8	12	0.0038 ± 0.0038	
ESR	0 ~ 20	30	0.0017 ± 0.0024	**0.0002**
	>20	28	0.0041 ± 0.0039	
D-Dimer	0.00 ~ 0.50	14	0.0023 ± 0.0029	0.052
	>0.5	21	0.0041 ± 0.0040	

* *C3/C4, complement 3/complement 4*.

*The bold values indicated P < 0.05*.

### hsa_circ_0000479 Regulates SLE Progression by Regulating Metabolic Pathways and the Wnt Signaling Pathway

**Figure 5:**
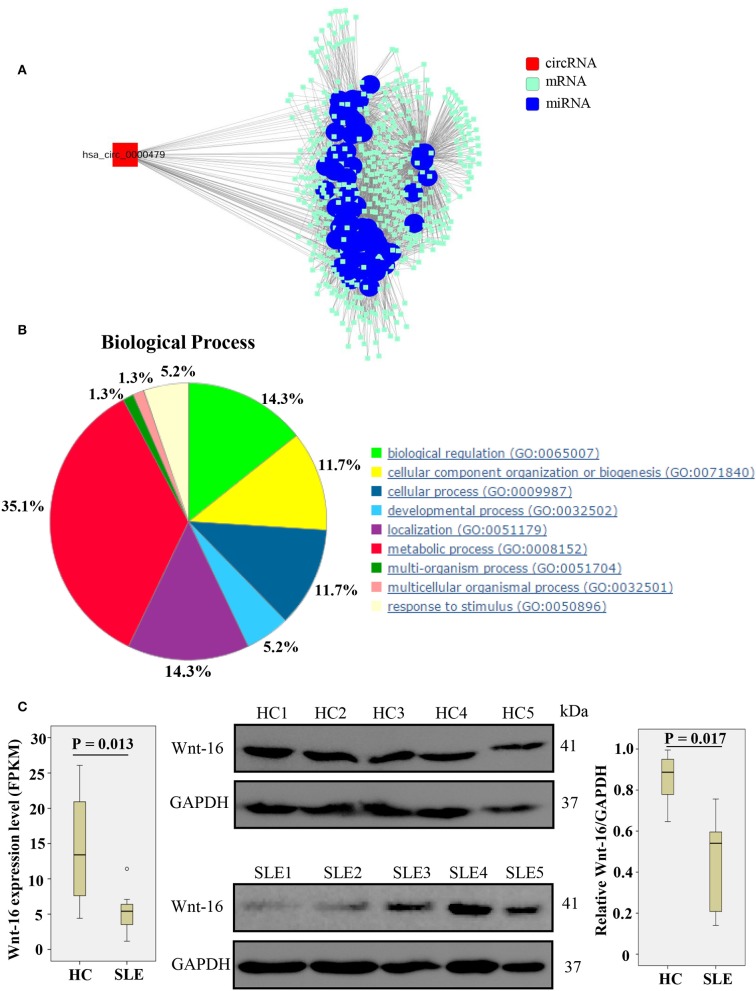
Biological functions of hsa_circ_0000479 acting as a ceRNA. **(A)** Predicted circRNA-miRNA network. The SLE-related miRNA is annotated by blue nodes. **(B)** Biological processes associated with the target genes of hsa_circ_0000479. **(C)** Wnt-16 mRNA and protein expression in PBMCs from SLE patients and healthy controls. Wnt-16 mRNA expression was evaluated by NGS-Seq in the discovery phase. Wnt-16 protein expression levels were normalized to those of GAPDH.

We constructed a circRNA-miRNA-target gene network to investigate the ceRNA network of hsa_circ_0000479 (Figure 5A). SLE-related target genes linked to the circRNA (red nodes) are enumerated in Supplementary Table 5. To further understand the underlying molecular mechanism(s) of hsa_circ_0000479, its target genes were investigated using GO analysis and the Thomson Reuters database (Figure 5B, Supplementary Table 6). The results of biological process analysis showed that the 488 target genes of hsa_circ_0000479 play vital roles in metabolic processes (35.1%), biological regulation (14.3%), and localization (14.3%). The Thomson Reuters database analysis showed that most relevant networks of hsa_circ_0000479's target genes are involved in regulation of cell proliferation (80.9%), regulation of the Wnt signaling pathway (51.1%), and the canonical Wnt signaling pathway (46.8%). These Wnt-related networks were the top-scoring network in most relevant networks (Supplementary Table 6). Meanwhile, the top-scoring (by the number of pathways) AN network of hsa_circ_0000479's target genes, established using the Thomson Reuters database, was the Wnt-related signaling pathway (Supplementary Figure 2, Supplementary Table 6).

According to our NGS results on differentially expressed mRNA, Wnt-16, a key member in the Wnt family (22), was downregulated (Supplementary Table 5, Figure 5C). To confirm further whether hsa_circ_0000479 downregulates the protein expression of Wnt-16, western blotting was performed. This showed significantly decreased expression of Wnt-16 protein in PBMCs of SLE patients compared with PBMCs from HCs (Figure 5C). These findings indicate that, compared HCs, the expression of Wnt-16 in PBMCs of SLE patients was downregulated at both the mRNA and protein levels.

## Discussion

This study constitutes the first multi-layered, comprehensive analysis using circRNA and mRNA expression profiles from PBMCs of SLE patients and healthy controls. We identified 114 aberrantly expressed circRNAs, of which 84 were upregulated and 30 were downregulated in SLE, with 93% originating from exons. Based on a classification of SLE disease activity, we further narrowed the candidates down to 15 circRNAs, all of which were upregulated in SLE patients. In contrast to a previous study using microarrays (7, 9, 11), we performed NGS analysis on circRNA and mRNA from PBMCs form SLE patients simultaneously. As a consequence, we identified a differentially expressed circRNA-mRNA network, which, to our knowledge has not previously been reported in SLE. KEGG analysis showed the target genes of our candidate circRNAs are enriched in metabolic pathways and oxidative phosphorylation, which are critical checkpoints of effector functions in SLE (20, 23). In addition, GO analysis showed they are involved in ATP-synthesis-coupled proton transport, a process which influences inflammatory and immune responses by mediating ATP level (24, 25).

Due to the complex clinical manifestations of SLE, the disease can be difficult to diagnose or distinguish from HCs. Furthermore, a single laboratory indication cannot provide a definitive SLE diagnosis. In light of our results, we therefore suggest that circRNAs may represent prospective biomarkers for diagnosis of SLE. Importantly, our ROC curve analysis quantified the potential clinical diagnostic value of hsa_circ_0000479 expression level for distinguishing SLE patients from HCs. Moreover, we also showed that qRT-PCR-validated hsa_circ_0000479 expression level can significantly distinguish between SLE patients and patients with RA. Further, analysis of *hsa_circ_0000479* expression among SLE-active patients, an SLE-stable group, and HCs revealed that expression of hsa_circ_0000479 can significantly distinguish between HCs and each of the two disease states in the validation-phase cohort and the external validation phase cohort. In addition to these analyses, we investigated the correlation between *hsa_circ_0000479* and clinical features of SLE patients. The results showed that the expression of hsa_circ_0000479 was positively correlated with anticardiolipin antibody IgG, high total IgG, and high ESR, which reflected the inflammatory and other immunomodulatory effects of the disease. High expression of hsa_circ_0000479 level was also correlated with low albumin level, positive urine protein, low leukocytes, and low hemoglobin, which indicated that it may be involved in renal injury and hematologic system damage. These results further indicated that *hsa_circ_0000479* is a novel promising diagnostic biomarker of SLE patients.

There has been a small number of previous reports of dysregulation of circRNAs in SLE patients. These data are not consistent across studies due to different samples and techniques. Li et al. screened 124 blood circRNAs in children with SLE and healthy donors using microarray profiling of circRNAs and mRNAs (9). Their qRT-PCR analysis showed increased expression of hsa_circ_0057762 and hsa_circ_0003090 in SLE, while expression of hsa_circ_0021372 and hsa_circ_0075699 were decreased. The level of hsa_circ_0057762 was positively correlated with SLEDAI and could distinguish SLE patients from HCs (AUC = 0.804). In addition, Zhang et al. screened 112 circRNAs differentially expressed in PBMCs of SLE patients compared with HCs by circRNA microarray (8). They found decreased expression of hsa_circRNA_407176 and hsa_circRNA_001308 in both PBMCs and plasma of SLE patients, compared with HCs, suggesting their potential as biomarkers for SLE (AUC = 0.806 and 0.722, respectively). Using circRNA expression profiles, Li et al. identified 127 dysregulated circRNAs in T cells of SLE patients and HCs (26). Among them, downregulation of hsa_circ_0045272, confirmed by qPCR, could negatively regulate apoptosis and interleukin-2 secretion. A significant advantage of our study against this background is that we confirmed the significant difference of hsa_circ_0000479 expression between SLE patients and HCs in three validation cohorts.

Upregulation of the circRNA *hsa_circ_0000479* differed significantly between SLE-active or SLE-stable patients and HCs, indicating that *hsa_circ_0000479* might be involved in SLE flare-ups. Moreover, our findings of a correlation between *hsa_circ_0000479* and clinical characteristics of SLE patients suggested that *hsa_circ_0000479* is associated with pathogenesis and organ damage in SLE. Based on bioinformatic analysis of its sequence, it is encoded by an exon, located at chromosome 13:43528084-43544806. Using the Thomson Reuters database and the analyze networks (AN) algorithm (21), we further investigated biological networks of its target genes. The most relevant networks were involved in the main regulation processes of SLE development, including the canonical Wnt signaling pathway, regulation of the Wnt pathway, and cell-cell signaling by Wnt. Compared with healthy controls, the results of our NGS results and western blotting further showed that the expression of Wnt-16 is downregulated in PBMCs of SLE patients, indicating that Wnt-16 may be involved in the pathogenesis of SLE. As reported, expression of Wnt proteins and aberrant Wnt-related signaling likely regulate metabolic processes and are involved in the potential pathological mechanism of chronic metabolic diseases (27). Wnt signaling plays pathogenic roles in several major autoimmune diseases, and ncRNAs targeting Wnt signaling in autoimmune disorders have shown potential as biomarkers and therapeutic targets, as well as associations with the development of autoimmune diseases (28). Wnt-16, one of a growing number of Wnt genes, is expressed widely, with highest levels in adult kidney, placenta, brain, heart, and spleen (29). A previous study verified that Wnt-16 can regulate p53 activity and the PI3K/AKT pathway, and is necessary for the onset of replicative senescence (22). Moreover, Wnt-16 has been shown to activate human keratinocyte proliferation (30). Furthermore, compared normal tissues, the expression of *Wnt-16* was upregulated in tumoral tissues, indicating that Wnt-16 may be associated with the progression of gastric cancer (31). Therefore, the abnormal expression of Wnt-16 we found suggests Wnt-related signaling may be involved in the pathogenesis of SLE. This further informs and motivates future exploration of the molecular mechanisms underlying the possible role of hsa_circ_0000479 in SLE progression.

Our study has several limitations. Firstly, our sample sizes were comparatively small. Replication with a larger sample size is clearly desirable to validate the results and to further explore whether hsa_circ_0000479 can reliably distinguish between SLE patients and HCs. Secondly, thorough evaluation of the specificity of the biomarker would require the inclusion of patients with additional inflammatory diseases. Thirdly, except for bioinformatics analysis, evidence on the molecular mechanisms of circRNAs in SLE is currently lacking.

## Conclusion

In summary, our circRNA and mRNA expression profiling in PBMCs from SLE patients and HCs revealed differentially expressed circRNAs. We further assessed the intersection of these aberrantly expressed circRNAs among SLE-active, SLE-stable, and HC groups to narrow down candidate circRNAs to 15, and identified *hsa_circ_0000479* as an outstanding potential diagnostic biomarker. In view of the regulatory links of this circRNA, these results may also help to elucidate the pathogenesis of SLE. Future investigation is needed to illuminate the roles of the circRNAs in SLE, and this work should eventually contribute to its diagnosis and treatment.

## Data Availability Statement

The raw data supporting the conclusions of this manuscript will be made available by the authors, without undue reservation, to any qualified researcher.

## Ethics Statement

The research protocol was approved by the Medical Ethical Committees of the First/Second Affiliated Hospital of Wenzhou Medical University and Renji Hospital of Shanghai Jiao Tong University of Medicine. All subjects who participated in this research provided written informed consent.

## Author Contributions

GG, HW, and LY performed the experiments, analyzed, and interpreted the data. GG and HW drafted the manuscript. XS performed the experiments and statistical analysis. KY, KL, QH, BL, QL, and LZ acquired the data and provided material support. XX and HZ contributed to the conception and design of the study, analyzed and interpreted the data, supervised the study, provided the project funding, revised the manuscript, and finally approved the version of the manuscript for publication. All authors read and approved the final manuscript.

### Conflict of Interest

The authors declare that the research was conducted in the absence of any commercial or financial relationships that could be construed as a potential conflict of interest.

## Supplementary Material

Supplementary Figure 1 Data analysis overview. Differentially expressed circRNAs (fold change >2 and *P* < 0.05). The intersection of these differentially expressed circRNAs among the SLE-stable, SLE-active, and HC groups was analyzed to narrow dysregulated circRNAs down to 15 upregulated circRNAs. Candidate circRNAs were then validated using qPCR in three cohorts. Finally, the association between candidate circRNAs in PBMCs and clinical characteristics of SLE patients were analyzed.

Supplementary Figure 2 The top-scoring (by the number of pathways) AN network from hsa_circ_0000479_target genes. Thick pink lines indicate fragments of WNT-related signaling pathways. Upregulated genes are marked with red circles; downregulated with blue circles. The red arrow indicates the hsa_circ_0000479 target gene *WNT-16*.

Supplementary Table 1 Clinical characteristics of SLE patients, RA patients, and healthy controls.

Supplementary Table 2 Primers used for qRT-PCR.

Supplementary Table 3 Differentially expressed circRNAn in PBMCs of SLE patients and normal controls.

Supplementary Table 4 The overlapping significantly changed circRNAs in PBMCs of SLE patients and normal controls.

Supplementary Table 5 The prediction of target gene of has-circ-0000497 ceRNA.

Supplementary Table 6 Most relevant networks of the target gene of circRNAs.
