# Supplementary figures and images for: Hsa_circ_0000479 as a Novel Diagnostic Biomarker of Systemic Lupus Erythematosus
Source: Front Immunol. 2019 Sep 24;10:2281. doi: 10.3389/fimmu.2019.02281 (PMC6771011; doi:10.3389/fimmu.2019.02281)

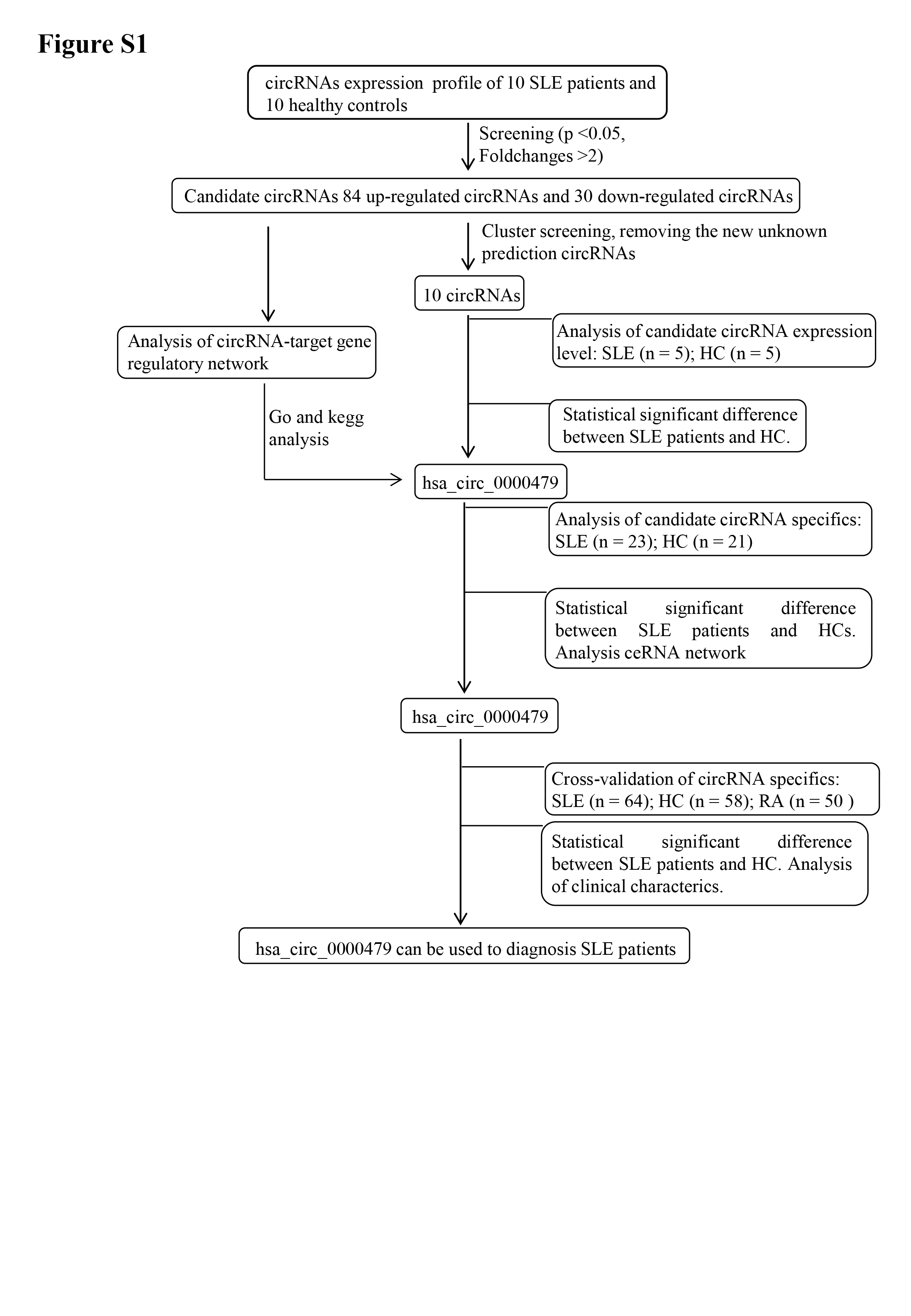

Supplement: Supplementary Figure 1 — Data analysis overview. Differentially expressed circRNAs (fold change >2 and P < 0.05). The intersection of these differentially expressed circRNAs among the SLE-stable, SLE-active, and HC groups was analyzed to narrow dysregulated circRNAs down to 15 upregulated circRNAs. Candidate circRNAs were then validated using qPCR in three cohorts. Finally, the association between candidate circRNAs in PBMCs and clinical characteristics of SLE patients were analyzed. [file Image_1.TIF]

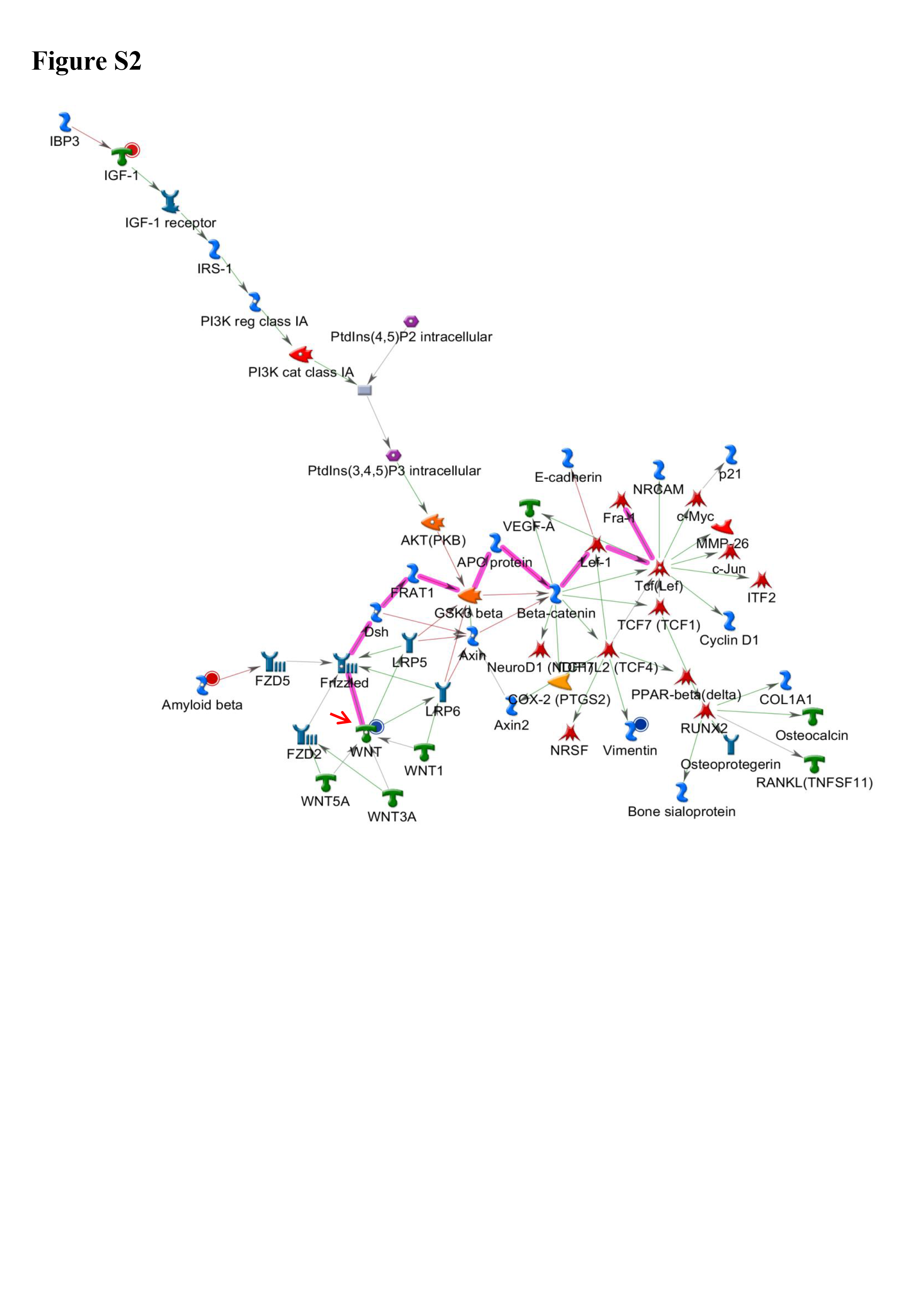

Supplement: Supplementary Figure 2 — The top-scoring (by the number of pathways) AN network from hsa_circ_0000479_target genes. Thick pink lines indicate fragments of WNT-related signaling pathways. Upregulated genes are marked with red circles; downregulated with blue circles. The red arrow indicates the hsa_circ_0000479 target gene WNT-16. [file Image_2.TIF]
