# Supplementary material for: Hsa_circ_0000479 as a Novel Diagnostic Biomarker of Systemic Lupus Erythematosus
Source: Front Immunol. 2019 Sep 24;10:2281. doi: 10.3389/fimmu.2019.02281 (PMC6771011; doi:10.3389/fimmu.2019.02281)
Supplement: Supplementary Table 1 — Clinical characteristics of SLE patients, RA patients, and healthy controls. [file Data_Sheet_1.docx]

| **Supplementary Table 1. Clinical characteristics of SLE patients, RA patients and healthy controls.** | | | | | | | |  |  | | | | | |  | |  | | | | | |  |  |
| --- | --- | --- | --- | --- | --- | --- | --- | --- | --- | --- | --- | --- | --- | --- | --- | --- | --- | --- | --- | --- | --- | --- | --- | --- |
| Clinical characteristics^#^ | Discovery phase (n=20) | | | | Training phase (n = 10) | | | | Validation phase (n = 44) | | | | | | | | External validation phase (n = 172) | | | | | | | |
|  | SLE (n=10) | | Healthy control (n=10) | | SLE (n=5) | | Healthy control (n=5) | | SLE (n = 23) | | | Healthy control (n=21) | | | | | SLE (n = 64) | | | Healthy control (n=58) | | | | Rheumatoid arthritis (n = 50) |
| Sex, male/female | 0/10 | | 0/10 | | 1/4 | | 1/4 | | 2/21 | | | 3/18 | | | | | 4/60 | | | 3/55 | | | | 16/34 |
| Age (year) | 40 ± 14.64 | | 30.60 ± 3.89 | | 37.20 ± 18.05 | | 35.62 ± 5.79 | | 37.65 ± 17.84 | | | 36.53 ± 10.60 | | | | | 35.98 ±12.89 | | | 37.55 ± 11.50 | | | | 52.68 ± 17.68 |
| Duration (year) | 5.80 ± 5.23 | |  | | 6.62 ± 4.30 | |  | | 5.79 ± 6.34 | | |  | | | | | 6.07 ± 6.19 | | |  | | | |  |
| SLEDAI scores | 10.00 ± 6.43 | |  | | 7.40 ± 5.13 | |  | | 7.0 ± 3.66 | | |  | | | | | 7.12 ± 4.76 | | |  | | | |  |
| disease active | 5(10) | |  | | 3(5) | |  | | 17 (23) | | |  | | | | | 32 (60) | | |  | | | |  |
| ANA (Positive) | 4 (4) | |  | | 4（5） | |  | | 4（22） | | |  | | | | | 41 (42) | | |  | | | |  |
| Anti-ds-DNA antibody | 3 (4) | |  | | 2（5） | |  | | 8（22） | | |  | | | | | 27 (58) | | |  | | | |  |
| Lupus anticoagulant | 1.26 ± 0.38 | |  | | 0.86 ± 0.21 | |  | | 1.04 ± 0.33 | | |  | | | | | 4.10 ± 17.13 | | |  | | | |  |
| Leukocyte | 5.56 ± 3.71 | |  | | 6.70 ± 3.53 | |  | | 6.50 ± 2.40 | | |  | | | | | 6.85 ± 4.72 | | |  | | | |  |
| Lymphocyte |  | |  | | 0.5 ± 0.45 | |  | | 1.21 ± 0.80 | | |  | | | | | 1.23 ± 0.77 | | |  | | | |  |
| Neutrophils | 4.03 ± 2.91 | |  | | 4.82 ± 2.67 | |  | | 4.85 ± 1.88 | | |  | | | | | 5.94 ± 11.29 | | |  | | | |  |
| Hemoglobin | 93.38 ± 13.65 | |  | | 100.00 ± 17.26 | |  | | 103.29 ± 20.25 | | |  | | | | | 104.20 ± 25.70 | | |  | | | |  |
| Platelets | 213.00 ± 39.71 | |  | | 162.60 ± 123.91 | |  | | 201.38 ± 79.65 | | |  | | | | | 185.72 ± 97.19 | | |  | | | |  |
| CRP |  | |  | | 5.0 ± 2.92 | |  | | 4.12 ± 4.11 | | |  | | | | | 10.65 ± 23.90 | | |  | | | |  |
| ESR |  | |  | | 27.40 ± 24.69 | |  | | 26.52 ±20.48 | | |  | | | | | 30.86 ± 30.78 | | |  | | | |  |
| ALT |  | |  | | 36.00 ± 41.44 | |  | | 32.19 ± 31.18 | | |  | | | | | 29.92 ± 28.61 | | |  | | | |  |
| AST |  | |  | | 29.20 ± 18.01 | |  | | 30.38 ± 19.39 | | |  | | | | | 29.20 ± 17.12 | | |  | | | |  |
| Serum creatinine | 62.56 ± 24.34 | |  | | 65.80 ± 25.84 | |  | | 60.54 ± 17.96 | | |  | | | | | 129.65 ± 187.47 | | |  | | | |  |
| Low C3 level | 5 (5) | |  | | 4（5） | |  | | 20（23） | | |  | | | | | 39 (59) | | |  | | | |  |
| Low C4 level | 5 (5) | |  | | 3（5） | |  | | 12（23） | | |  | | | | | 37 (57) | | |  | | | |  |
| 24h urine protein positive | 4 (5) | |  | | 1（5） | |  | | 11（23） | | |  | | | | | 30 (37) | | |  | | | |  |
| ^#^C3/C4 complement 3/complement 4; SLEDAI, systemic lupus erythematosus disease activity index; ALT, alanine aminotransferase | | | | | | | | | | | |  | | | | |  | |  | | | | |  |
| **Supplementary Table 2.Primers used for qRT-PCR.** | | | | | |  | | | | |  | | |  | | |  | | | |  |  |  |  |
| Gene Name | | Forward primer (5'-3') | | | | Reverse primer (5'-3') | | | | | TM (℃) | | | Product (bp) | | |  | | | |  |  |  |  |
| hsa_circ_0003764 | | GTTCCTATTTGTATTCATTGC | | | | TCGTTCTTGCTTGTTCAT | | | | | 58 | | | 156 | | |  | | | |  |  |  |  |
| hsa_circ_0004442 | | TTAGACTAGGACATCTCAA | | | | TTGGTTCAGAATTGGTAG | | | | | 58 | | | 142 | | |  | | | |  |  |  |  |
| hsa_circ_0004771 | | TTGGAGACAGACGGAAGT | | | | GCATCTTCTGGCTGTGTT | | | | | 58 | | | 97 | | |  | | | |  |  |  |  |
| hsa_circ_0006459 | | AAGTTCTCGTGGAGTCAG | | | | CACATCTTGTTGCGTAGG | | | | | 58 | | | 87 | | |  | | | |  |  |  |  |
| hsa_circ_0008139 | | GGATGATATGGGAGTATAATGTTG | | | | ATAGTCTGAATCTTGTGAAGGA | | | | | 58 | | | 82 | | |  | | | |  |  |  |  |
| hsa_circ_0018168 | | TCCAAACCCTCACCTGTA | | | | ATTCGTATCCTCTCCTCTTCT | | | | | 58 | | | 116 | | |  | | | |  |  |  |  |
| hsa_circ_0001449 | | GGAAGTGAAGACAATAACAAGT | | | | TCATTTATACTGGCTGGAACA | | | | | 58 | | | 111 | | |  | | | |  |  |  |  |
| hsa_circ_0000479 | | AAGAGAAGAATCTGTAAGAATCA | | | | TGGTGCTATCAAGGTGTA | | | | | 58 | | | 181 | | |  | | | |  |  |  |  |
| hsa_circ_0000437 | | AATGTGAGATTGCCAGAA | | | | GTCAGGGTCATAGAAAGG | | | | | 58 | | | 94 | | |  | | | |  |  |  |  |
| GAPDH | | CGGAGTCAACGGATTTGGTC | | | | CGGTGCCATGGAATTTGCCA | | | | | 58 | | | 161 | | |  | | | |  |  |  |  |
| **Supplementary Table 3. Differentially expressed circRNAn in PBMCs of SLE patients and normal controls.** | | | | | | | | | | | | | | | | | |  | | | |  |  |  |
| Gene name | | Type | | annotated_circRNA_ID | | | | | | log2FoldChange | | | pvalue | | | padj | |  | | | |  |  |  |
| chr12:103657104-103664086 | | exon | | N/A | | | | | | -2.190112229 | | | 0.000494276 | | | 0.01239352 | |  | | | |  |  |  |
| chr14:50130033-50141145 | | exon | | hsa_circ_0008002 | | | | | | 1.14790464 | | | 0.000114457 | | | 0.004304841 | |  | | | |  |  |  |
| chr17:26449629-26499644 | | exon | | hsa_circ_0002103 | | | | | | 1.12300036 | | | 0.001024025 | | | 0.020856917 | |  | | | |  |  |  |
| chr12:42745687-42792796 | | exon | | N/A | | | | | | 1.425320608 | | | 0.00039572 | | | 0.01043776 | |  | | | |  |  |  |
| chr11:128993341-129034322 | | exon | | N/A | | | | | | 2.186599262 | | | 6.59E-05 | | | 0.00280239 | |  | | | |  |  |  |
| chr8:62593527-62596747 | | exon | | N/A | | | | | | 1.446996172 | | | 0.000618915 | | | 0.014788436 | |  | | | |  |  |  |
| chr2:33442619-33447218 | | exon | | N/A | | | | | | 2.439184823 | | | 3.95E-05 | | | 0.002219031 | |  | | | |  |  |  |
| chr6:99860427-99864304 | | exon | | N/A | | | | | | -1.127131457 | | | 0.003307497 | | | 0.048476245 | |  | | | |  |  |  |
| chr9:37126309-37147442 | | exon | | N/A | | | | | | -1.043083828 | | | 0.001915493 | | | 0.032969198 | |  | | | |  |  |  |
| chr13:43528084-43544806 | | exon | | hsa_circ_0000479 | | | | | | 2.280340037 | | | 4.53E-09 | | | 2.30E-06 | |  | | | |  |  |  |
| chr7:65751498-65751696 | | exon | | N/A | | | | | | 1.97347332 | | | 2.72E-05 | | | 0.00162731 | |  | | | |  |  |  |
| chr6:3076998-3078169 | | exon | | N/A | | | | | | -1.310941322 | | | 4.89E-05 | | | 0.002423702 | |  | | | |  |  |  |
| chr19:23541232-23545527 | | exon | | - | | | | | | -1.511124943 | | | 5.62E-06 | | | 0.000438834 | |  | | | |  |  |  |
| chr2:33525518-33526711 | | exon | | N/A | | | | | | 2.163792271 | | | 0.000308076 | | | 0.009068162 | |  | | | |  |  |  |
| chr2:171884849-171902872 | | exon | | hsa_circ_0004442 | | | | | | 1.522019521 | | | 3.57E-07 | | | 4.26E-05 | |  | | | |  |  |  |
| chr11:128638013-128651918 | | exon | | N/A | | | | | | 1.223031194 | | | 0.002096334 | | | 0.035477394 | |  | | | |  |  |  |
| chr2:203620261-203624076 | | exon | | N/A | | | | | | -1.865246681 | | | 0.000185011 | | | 0.006159968 | |  | | | |  |  |  |
| chr15:66044717-66053776 | | exon | | hsa_circ_0035957 | | | | | | 1.197558987 | | | 4.82E-05 | | | 0.002423702 | |  | | | |  |  |  |
| chr12:45771836-45784281 | | exon | | hsa_circ_0005192 | | | | | | 1.738650198 | | | 2.83E-06 | | | 0.000252889 | |  | | | |  |  |  |
| chr12:68696400-68696652 | | exon | | hsa_circ_0027459 | | | | | | 1.219606948 | | | 0.000372898 | | | 0.010272031 | |  | | | |  |  |  |
| chr1:205585606-205593019 | | exon | | hsa_circ_0000175 | | | | | | -1.177898433 | | | 1.84E-07 | | | 2.69E-05 | |  | | | |  |  |  |
| chrX:17121841-17157084 | | exon | | N/A | | | | | | 1.544182336 | | | 0.001795172 | | | 0.031162343 | |  | | | |  |  |  |
| chrX:64271342-64295792 | | intergenic_region | | hsa_circ_0001924 | | | | | | -1.54507775 | | | 1.25E-07 | | | 2.14E-05 | |  | | | |  |  |  |
| chr1:86590528-86611866 | | exon | | N/A | | | | | | 1.631780404 | | | 0.001496867 | | | 0.027388619 | |  | | | |  |  |  |
| chr13:96375496-96377506 | | exon | | N/A | | | | | | 1.521796026 | | | 7.01E-05 | | | 0.002906595 | |  | | | |  |  |  |
| chr8:110422094-110432919 | | exon | | - | | | | | | 1.651314071 | | | 1.89E-05 | | | 0.001236921 | |  | | | |  |  |  |
| chr10:24918925-24924032 | | exon | | hsa_circ_0017988,hsa_circ_0017989,hsa_circ_0017990, | | | | | | 3.163811111 | | | 2.14E-10 | | | 2.17E-07 | |  | | | |  |  |  |
| chr6:90959408-90981660 | | intergenic_region | | N/A | | | | | | -2.119361272 | | | 0.000189371 | | | 0.006203428 | |  | | | |  |  |  |
| chr14:50136242-50141145 | | exon | | N/A | | | | | | 1.758665674 | | | 0.000733747 | | | 0.016198267 | |  | | | |  |  |  |
| chr6:89793472-89794362 | | exon | | - | | | | | | -1.430933133 | | | 6.66E-07 | | | 7.12E-05 | |  | | | |  |  |  |
| chr7:151946961-151948051 | | exon | | N/A | | | | | | 1.707006494 | | | 7.29E-06 | | | 0.000528702 | |  | | | |  |  |  |
| chr16:47531310-47581459 | | exon | | hsa_circ_0004791 | | | | | | 1.338709975 | | | 0.000457535 | | | 0.011762705 | |  | | | |  |  |  |
| chr21:38792601-38794168 | | intron | | N/A | | | | | | 1.497728308 | | | 0.000327964 | | | 0.009496049 | |  | | | |  |  |  |
| chr10:98667022-98667504 | | exon | | N/A | | | | | | 1.206471839 | | | 0.000393026 | | | 0.01043776 | |  | | | |  |  |  |
| chr17:26490569-26499644 | | exon | | hsa_circ_0003638 | | | | | | 1.24379745 | | | 5.71E-07 | | | 6.45E-05 | |  | | | |  |  |  |
| chr15:93540187-93541851 | | exon | | N/A | | | | | | -1.538447798 | | | 0.000565516 | | | 0.013673362 | |  | | | |  |  |  |
| chr16:58594116-58594266 | | exon | | N/A | | | | | | 1.748386723 | | | 0.001676665 | | | 0.029611355 | |  | | | |  |  |  |
| chr2:15691617-15698758 | | exon | | N/A | | | | | | 1.617067159 | | | 0.003271505 | | | 0.048476245 | |  | | | |  |  |  |
| chr9:114840818-114842445 | | exon | | N/A | | | | | | 1.33823153 | | | 0.000692473 | | | 0.015455083 | |  | | | |  |  |  |
| chr15:49528048-49531564 | | exon | | N/A | | | | | | 1.14436721 | | | 0.001066708 | | | 0.021356413 | |  | | | |  |  |  |
| chr11:120276827-120278532 | | exon | | N/A | | | | | | 1.652996676 | | | 6.85E-06 | | | 0.000515082 | |  | | | |  |  |  |
| chr3:16343133-16344255 | | exon | | N/A | | | | | | -1.615233864 | | | 0.000207729 | | | 0.006696792 | |  | | | |  |  |  |
| chr21:16412684-16415895 | | intergenic_region | | N/A | | | | | | 2.282550175 | | | 2.86E-05 | | | 0.001657035 | |  | | | |  |  |  |
| chr7:77210744-77214898 | | exon | | N/A | | | | | | 1.61749602 | | | 0.001234202 | | | 0.023647773 | |  | | | |  |  |  |
| chr6:111067329-111095140 | | exon | | N/A | | | | | | 1.688314769 | | | 0.002578649 | | | 0.040915905 | |  | | | |  |  |  |
| chr3:17051166-17056403 | | exon | | hsa_circ_0001274 | | | | | | -1.923862389 | | | 0.000179755 | | | 0.006102146 | |  | | | |  |  |  |
| chr18:43568668-43573669 | | exon | | N/A | | | | | | 1.457560605 | | | 0.000532211 | | | 0.013023147 | |  | | | |  |  |  |
| chr13:78293667-78327493 | | exon | | hsa_circ_0000497 | | | | | | -1.308515766 | | | 8.63E-05 | | | 0.003435885 | |  | | | |  |  |  |
| chr1:79132257-79138776 | | intergenic_region | | N/A | | | | | | 5.246698911 | | | 0.002334734 | | | 0.038240682 | |  | | | |  |  |  |
| chr4:17816476-17816981 | | exon | | N/A | | | | | | 2.016950838 | | | 4.04E-05 | | | 0.002219031 | |  | | | |  |  |  |
| chr16:68224671-68225678 | | exon | | N/A | | | | | | -1.553121623 | | | 0.000649519 | | | 0.014976363 | |  | | | |  |  |  |
| chr9:114860750-114875148 | | exon | | N/A | | | | | | 1.502271902 | | | 0.002326148 | | | 0.038240682 | |  | | | |  |  |  |
| chr14:73614503-73614814 | | exon | | N/A | | | | | | 1.571210228 | | | 0.000639012 | | | 0.014976363 | |  | | | |  |  |  |
| chr4:148743878-148744108 | | exon | | N/A | | | | | | 1.944021763 | | | 2.60E-07 | | | 3.52E-05 | |  | | | |  |  |  |
| chr18:76953183-76974038 | | exon | | N/A | | | | | | 1.174230575 | | | 0.001026928 | | | 0.020856917 | |  | | | |  |  |  |
| chr6:117010483-117026323 | | exon | | N/A | | | | | | -1.400294933 | | | 0.003009851 | | | 0.045619455 | |  | | | |  |  |  |
| chr7:77214860-77230123 | | exon | | hsa_circ_0003764 | | | | | | 1.035638888 | | | 3.06E-06 | | | 0.000259017 | |  | | | |  |  |  |
| chr4:148860976-148876525 | | exon | | hsa_circ_0001449 | | | | | | 1.544884801 | | | 3.04E-07 | | | 3.85E-05 | |  | | | |  |  |  |
| chr13:43537391-43544806 | | exon | | N/A | | | | | | 2.776390291 | | | 5.34E-05 | | | 0.00252342 | |  | | | |  |  |  |
| chr19:41847788-41848152 | | exon | | N/A | | | | | | 1.484310575 | | | 0.002838158 | | | 0.043668932 | |  | | | |  |  |  |
| chr3:71090479-71102924 | | exon | | N/A | | | | | | -1.115526081 | | | 0.001744688 | | | 0.030547088 | |  | | | |  |  |  |
| chr21:16386665-16417644 | | intergenic_region | | N/A | | | | | | 1.131222453 | | | 0.002628357 | | | 0.041063023 | |  | | | |  |  |  |
| chr16:47531310-47549512 | | exon | | N/A | | | | | | 1.233857425 | | | 0.000304717 | | | 0.009068162 | |  | | | |  |  |  |
| chr1:21415631-21437876 | | exon | | N/A | | | | | | 1.74352335 | | | 0.002789259 | | | 0.043244156 | |  | | | |  |  |  |
| chr4:151656410-151729550 | | exon | | N/A | | | | | | 1.275257551 | | | 0.002865687 | | | 0.043760987 | |  | | | |  |  |  |
| chr9:80409379-80412564 | | exon | | hsa_circ_0006459 | | | | | | 1.610161872 | | | 1.85E-07 | | | 2.69E-05 | |  | | | |  |  |  |
| chr10:54031110-54050050 | | exon | | N/A | | | | | | 2.237410467 | | | 0.000392123 | | | 0.01043776 | |  | | | |  |  |  |
| chr10:74474869-74475660 | | exon | | N/A | | | | | | 2.680163025 | | | 2.86E-06 | | | 0.000252889 | |  | | | |  |  |  |
| chr7:77210744-77221573 | | exon | | hsa_circ_0002458 | | | | | | 1.040983288 | | | 0.000100233 | | | 0.003914888 | |  | | | |  |  |  |
| chr3:71064700-71102924 | | exon | | N/A | | | | | | -1.782288895 | | | 0.00082442 | | | 0.017529129 | |  | | | |  |  |  |
| chr9:88918001-88919862 | | exon | | N/A | | | | | | 1.238294088 | | | 0.000501785 | | | 0.012428358 | |  | | | |  |  |  |
| chr8:42761316-42785315 | | exon | | N/A | | | | | | 1.133686688 | | | 0.002388327 | | | 0.038805535 | |  | | | |  |  |  |
| chr12:46633462-46637097 | | exon | | N/A | | | | | | -1.410517339 | | | 9.14E-06 | | | 0.000640399 | |  | | | |  |  |  |
| chr22:32154532-32161046 | | exon | | N/A | | | | | | 2.293592825 | | | 4.17E-06 | | | 0.00033891 | |  | | | |  |  |  |
| chr10:34558585-34573173 | | exon | | hsa_circ_0018168 | | | | | | 1.936514053 | | | 9.06E-08 | | | 1.84E-05 | |  | | | |  |  |  |
| chr3:51575514-51586079 | | intron | | hsa_circ_0001306 | | | | | | -1.159573441 | | | 4.25E-05 | | | 0.002273567 | |  | | | |  |  |  |
| chr12:25232178-25236427 | | exon | | N/A | | | | | | 1.892887171 | | | 2.63E-05 | | | 0.001620421 | |  | | | |  |  |  |
| chr9:80409379-80537261 | | exon | | N/A | | | | | | 1.366294069 | | | 0.000331964 | | | 0.009496049 | |  | | | |  |  |  |
| chr18:39623697-39629569 | | exon | | N/A | | | | | | 1.920347862 | | | 0.000919679 | | | 0.01925637 | |  | | | |  |  |  |
| chr17:27809215-27818883 | | exon | | hsa_circ_0007882 | | | | | | 1.395466251 | | | 5.80E-05 | | | 0.002559928 | |  | | | |  |  |  |
| chr8:131164982-131193126 | | exon | | hsa_circ_0008934 | | | | | | 1.128440556 | | | 1.83E-05 | | | 0.001236921 | |  | | | |  |  |  |
| chr2:65318117-65325200 | | exon | | N/A | | | | | | 1.856744539 | | | 0.001962172 | | | 0.033488827 | |  | | | |  |  |  |
| chr7:148543562-148544397 | | exon | | N/A | | | | | | 1.580750312 | | | 0.000484922 | | | 0.012310968 | |  | | | |  |  |  |
| chr10:16794538-16824083 | | exon | | hsa_circ_0006577 | | | | | | 1.283387204 | | | 1.19E-08 | | | 4.84E-06 | |  | | | |  |  |  |
| chr7:77210744-77212967 | | exon | | hsa_circ_0008139 | | | | | | 1.649716011 | | | 1.86E-08 | | | 6.29E-06 | |  | | | |  |  |  |
| chr10:74468041-74475660 | | exon | | N/A | | | | | | 1.375605922 | | | 0.000288327 | | | 0.008740186 | |  | | | |  |  |  |
| chr11:108172375-108175579 | | exon | | N/A | | | | | | -1.387363663 | | | 0.000656276 | | | 0.014976363 | |  | | | |  |  |  |
| chr13:43500472-43538275 | | exon | | N/A | | | | | | 2.821986645 | | | 0.000237907 | | | 0.007549838 | |  | | | |  |  |  |
| chr4:56277781-56284152 | | exon | | hsa_circ_0001414 | | | | | | 1.000847979 | | | 0.000140016 | | | 0.004989008 | |  | | | |  |  |  |
| chr8:106431372-106456609 | | exon | | N/A | | | | | | 1.871945254 | | | 6.62E-05 | | | 0.00280239 | |  | | | |  |  |  |
| chrX:100296306-100297301 | | exon | | N/A | | | | | | -1.274628408 | | | 0.000125125 | | | 0.004538016 | |  | | | |  |  |  |
| chr14:99723808-99724176 | | exon | | N/A | | | | | | -1.054809812 | | | 0.000645938 | | | 0.014976363 | |  | | | |  |  |  |
| chr2:191927488-191931241 | | exon | | N/A | | | | | | -1.8235749 | | | 5.27E-05 | | | 0.00252342 | |  | | | |  |  |  |
| chr13:43491677-43544806 | | exon | | N/A | | | | | | 2.358956237 | | | 1.20E-06 | | | 0.000121543 | |  | | | |  |  |  |
| chr12:46622936-46637097 | | exon | | hsa_circ_0000396 | | | | | | -1.120127711 | | | 3.65E-08 | | | 8.44E-06 | |  | | | |  |  |  |
| chr8:618598-624047 | | exon | | N/A | | | | | | -1.617694688 | | | 2.43E-05 | | | 0.001539217 | |  | | | |  |  |  |
| chr4:42505467-42546003 | | exon | | N/A | | | | | | 1.323041739 | | | 0.003053145 | | | 0.045932869 | |  | | | |  |  |  |
| chr11:14793483-14810788 | | exon | | N/A | | | | | | -1.182316071 | | | 0.001635786 | | | 0.029142817 | |  | | | |  |  |  |
| chr1:176012322-176015460 | | exon | | N/A | | | | | | -1.983300871 | | | 0.000447324 | | | 0.011647639 | |  | | | |  |  |  |
| chr2:11426665-11427862 | | exon | | N/A | | | | | | 1.364351736 | | | 0.001608439 | | | 0.028909196 | |  | | | |  |  |  |
| chr17:81042814-81043199 | | exon | | N/A | | | | | | -2.108508692 | | | 0.00018027 | | | 0.006102146 | |  | | | |  |  |  |
| chr21:16386665-16415895 | | exon | | hsa_circ_0004771 | | | | | | 1.682578088 | | | 6.98E-16 | | | 1.42E-12 | |  | | | |  |  |  |
| chr17:46187329-46195399 | | intron | | - | | | | | | -1.487950916 | | | 5.71E-05 | | | 0.002559928 | |  | | | |  |  |  |
| chr10:52193236-52350007 | | exon | | N/A | | | | | | 1.140416908 | | | 0.002113621 | | | 0.035477394 | |  | | | |  |  |  |
| chr12:109046048-109048186 | | exon | | hsa_circ_0000437 | | | | | | 1.139330339 | | | 1.27E-07 | | | 2.14E-05 | |  | | | |  |  |  |
| chr22:17117930-17119630 | | exon | | hsa_circ_0062137,hsa_circ_0062139,hsa_circ_0062141,hsa_circ_0062142, | | | | | | 1.651696763 | | | 3.03E-08 | | | 8.44E-06 | |  | | | |  |  |  |
| chr16:47345151-47347734 | | exon | | N/A | | | | | | 1.90371172 | | | 0.001412822 | | | 0.026568907 | |  | | | |  |  |  |
| chr14:66028055-66028484 | | exon | | N/A | | | | | | 1.443777505 | | | 0.003285691 | | | 0.048476245 | |  | | | |  |  |  |
| chr9:99284788-99327765 | | exon | | N/A | | | | | | 1.575622476 | | | 0.000355713 | | | 0.010034058 | |  | | | |  |  |  |
| chr13:43500472-43544806 | | exon | | hsa_circ_0000479,hsa_circ_0030145, | | | | | | 2.825774305 | | | 2.83E-09 | | | 1.91E-06 | |  | | | |  |  |  |
| chr18:756557-756835 | | exon | | N/A | | | | | | 1.561472558 | | | 0.000170516 | | | 0.005971012 | |  | | | |  |  |  |
| chr18:43577715-43579500 | | exon | | N/A | | | | | | 1.923355054 | | | 0.000684295 | | | 0.015442254 | |  | | | |  |  |  |
| chr11:124517261-124518071 | | exon | | hsa_circ_0000367 | | | | | | 1.463704033 | | | 1.71E-06 | | | 0.000165451 | |  | | | |  |  |  |
| chr7:77210744-77230123 | | exon | | N/A | | | | | | 1.127969247 | | | 0.0015877 | | | 0.028791235 | |  | | | |  |  |  |

| **Supplementary Table 4.The overlapping significantly changed circRNAs in PBMCs of SLE patients and normal controls.** | | | | |  |
| --- | --- | --- | --- | --- | --- |
| Gene name | log2FoldChange | pvalue | padj | annotated_circRNA_ID | Selected |
| chr4:148860976-148876525 | -1.544884801 | 3.03573E-07 | 3.8535E-05 | hsa_circ_0001449 | √ |
| chr13:43491677-43544806 | -2.358956237 | 1.19687E-06 | 0.00012154 | N/A |  |
| chr13:43500472-43544806 | -2.825774305 | 2.82687E-09 | 1.9138E-06 | hsa_circ_0000479, hsa_circ_0030145 |  |
| chr4:148743878-148744108 | -1.944021763 | 2.60232E-07 | 3.5235E-05 | N/A |  |
| chr7:77210744-77212967 | -1.649716011 | 1.85774E-08 | 6.2885E-06 | hsa_circ_0008139 | √ |
| chr7:65751498-65751696 | -1.97347332 | 2.7242E-05 | 0.00162731 | N/A |  |
| chr2:171884849-171902872 | -1.522019521 | 3.56637E-07 | 4.2608E-05 | hsa_circ_0004442 | √ |
| chr12:109046048-109048186 | -1.139330339 | 1.26685E-07 | 2.1441E-05 | hsa_circ_0000437 | √ |
| chr10:24918925-24924032 | -3.163811111 | 2.14177E-10 | 2.175E-07 | hsa_circ_0017988, hsa_circ_0017989, hsa_circ_0017990 | |
| chr13:43528084-43544806 | -2.280340037 | 4.53123E-09 | 2.3007E-06 | hsa_circ_0000479 | √ |
| chr9:80409379-80412564 | -1.610161872 | 1.85361E-07 | 2.6891E-05 | hsa_circ_0006459 | √ |
| chr7:77214860-77230123 | -1.035638888 | 3.06076E-06 | 0.00025902 | hsa_circ_0003764 | √ |
| chr21:16386665-16415895 | -1.682578088 | 6.97814E-16 | 1.4173E-12 | hsa_circ_0004771 | √ |
| chr12:45771836-45784281 | -1.738650198 | 2.83091E-06 | 0.00025289 | hsa_circ_0005192 | √ |
| chr10:34558585-34573173 | -1.936514053 | 9.05629E-08 | 1.8393E-05 | hsa_circ_0018168 | √ |

| **Supplementary Table 5. The prediction of target gene of has-circ-0000497 ceRNA.** | | | | |
| --- | --- | --- | --- | --- |
| target gene | GeneSymbol | log2FoldChange | pvalue | Regulation |
| ENST00000608642 | LARGE | 1.226327761 | 0.001899184 | Up |
| ENST00000607453 | RP11-156E8.1 | -1.783908563 | 3.20099E-10 | Down |
| ENST00000607000 | SERAC1 | 1.161970937 | 0.002602543 | Up |
| ENST00000606911 | TOR1AIP1 | 1.331969527 | 7.98275E-06 | Up |
| ENST00000606017 | CD24 | 1.07431026 | 0.002287908 | Up |
| ENST00000605787 | RP11-331G2.6 | 1.041647865 | 0.000397474 | Up |
| ENST00000602910 | SMOX | 1.424810464 | 0.00313193 | Up |
| ENST00000602840 | TCEB1 | 1.136025233 | 0.000013453 | Up |
| ENST00000602321 | RLTPR | -1.794839489 | 0.002962188 | Down |
| ENST00000601984 | EVI5L | 1.433864614 | 0.000702084 | Up |
| ENST00000601424 | AC002451.1 | 1.576143885 | 8.23832E-07 | Up |
| ENST00000601375 | OR7E94P | 1.089107905 | 0.000413172 | Up |
| ENST00000600235 | CPAMD8 | 1.656685615 | 0.003618499 | Up |
| ENST00000599790 | EPS15L1 | -1.384802739 | 0.000768122 | Down |
| ENST00000597013 | ZNF616 | 1.582705645 | 0.002797552 | Up |
| ENST00000596396 | AL590452.1 | -2.026414348 | 0.002704868 | Down |
| ENST00000596294 |  | -1.10374059 | 0.001089701 | Down |
| ENST00000596242 | STAP2 | 1.222817067 | 0.002160523 | Up |
| ENST00000596217 | NUP62 | 1.714143574 | 2.83284E-06 | Up |
| ENST00000595101 | USE1 | 1.817125116 | 2.08248E-06 | Up |
| ENST00000594821 | ZNF431 | 1.230875148 | 3.85381E-05 | Up |
| ENST00000593687 | ZNF546 | 1.485442461 | 5.56741E-05 | Up |
| ENST00000593019 | RBFA | 1.081102792 | 3.91204E-08 | Up |
| ENST00000592988 | AFMID | 1.175816107 | 6.75079E-05 | Up |
| ENST00000592551 | C19orf66 | 1.278596684 | 0.000198459 | Up |
| ENST00000592495 | ZNF439 | 2.198108667 | 0.000765121 | Up |
| ENST00000590993 | ZFP82 | 1.077045769 | 0.000283676 | Up |
| ENST00000590602 | TMC6 | -1.006290081 | 8.36642E-05 | Down |
| ENST00000589889 | SEMA6B | 1.593752452 | 4.57523E-11 | Up |
| ENST00000589056 | PIK3C3 | 1.405696484 | 0.001059334 | Up |
| ENST00000588534 | ZNF554 | 1.085937505 | 5.79089E-06 | Up |
| ENST00000587856 | FMNL1 | -1.431600503 | 4.44602E-06 | Down |
| ENST00000587787 | FAM198B | 1.560862431 | 0.002784911 | Up |
| ENST00000587491 | SEC14L1 | 1.401766458 | 1.90119E-05 | Up |
| ENST00000587309 | KIF18B | 1.495664491 | 0.000274849 | Up |
| ENST00000586801 | CNTNAP1 | -1.392616215 | 0.001646426 | Down |
| ENST00000586009 | CELF4 | -1.464067655 | 0.000657481 | Down |
| ENST00000585307 | THOC1 | -2.053715543 | 3.77307E-06 | Down |
| ENST00000584658 | SLC5A10 | -1.015356259 | 0.002677528 | Down |
| ENST00000584580 | LRRC46 | -1.94848131 | 0.001516215 | Down |
| ENST00000584501 | STAC2 | 1.767824281 | 8.20788E-08 | Up |
| ENST00000584259 | ENOSF1 | 2.017393424 | 1.14503E-05 | Up |
| ENST00000583418 | CYB5A | 1.010735839 | 0.002031381 | Up |
| ENST00000583301 | NSRP1 | 1.223288845 | 7.67356E-07 | Up |
| ENST00000582783 | C19orf47 | 1.310869384 | 0.002070416 | Up |
| ENST00000582019 | ASPSCR1 | 1.266304301 | 0.000440974 | Up |
| ENST00000581692 | BZRAP1 | 2.68266453 | 6.33112E-07 | Up |
| ENST00000581407 | FAM222B | -1.48532882 | 0.001615753 | Down |
| ENST00000579489 | MYH3 | 1.692501144 | 0.001929186 | Up |
| ENST00000579225 | SRGAP2 | 1.76377056 | 0.00023516 | Up |
| ENST00000579147 | PSMC5 | -1.143837427 | 0.000373668 | Down |
| ENST00000578846 | DUS1L | -1.151806298 | 0.002265007 | Down |
| ENST00000577583 | TAOK1 | 1.415702725 | 0.001043328 | Up |
| ENST00000576157 |  | 1.059939939 | 0.003417311 | Up |
| ENST00000575079 | PSMB6 | 2.134043747 | 2.57056E-05 | Up |
| ENST00000574259 |  | 1.351376181 | 0.002409826 | Up |
| ENST00000573956 | ANAPC11 | -1.258019191 | 0.000209308 | Down |
| ENST00000573729 |  | -3.198294416 | 0.000117985 | Down |
| ENST00000573483 | CAMKK1 | 1.107195487 | 0.000365419 | Up |
| ENST00000573376 | SAMD14 | -1.04966353 | 6.53729E-06 | Down |
| ENST00000572994 |  | 1.059939939 | 0.003417311 | Up |
| ENST00000572687 | PAQR4 | 2.201850783 | 0.000293606 | Up |
| ENST00000572585 | ABR | -1.029855966 | 0.001894185 | Down |
| ENST00000572400 | MKL2 | 1.437505839 | 0.000451406 | Up |
| ENST00000571346 | PRPF8 | 1.027156573 | 0.000388832 | Up |
| ENST00000569466 | CD2BP2 | 1.524210141 | 0.00413369 | Up |
| ENST00000569183 | WDR59 | 1.42034302 | 0.002678312 | Up |
| ENST00000569108 | SPIRE2 | 1.127953907 | 0.001704339 | Up |
| ENST00000568490 | TYRO3 | 1.161613784 | 0.000798534 | Up |
| ENST00000568064 | ZNF276 | 1.191810282 | 3.25371E-05 | Up |
| ENST00000566894 | FAM219B | -1.37598689 | 0.003610616 | Down |
| ENST00000566882 | CCNDBP1 | 1.22947128 | 0.002695301 | Up |
| ENST00000566692 |  | 1.558713189 | 0.002326509 | Up |
| ENST00000565951 | CPNE2 | -1.077541974 | 0.000874344 | Down |
| ENST00000565749 | ACKR2 | 1.865162127 | 0.000418694 | Up |
| ENST00000564238 | SPATA33 | 1.302049755 | 0.000851998 | Up |
| ENST00000564161 | ZC3H18 | 1.329487727 | 0.001400178 | Up |
| ENST00000564068 | COMMD4 | 1.318257102 | 0.004042581 | Up |
| ENST00000563604 | CDAN1 | -1.169447486 | 0.002588959 | Down |
| ENST00000563309 |  | -1.576523586 | 0.000469093 | Down |
| ENST00000561976 | VPS9D1 | -1.829511325 | 0.002272717 | Down |
| ENST00000561733 | RBMX | 1.756740408 | 3.60366E-07 | Up |
| ENST00000561213 | CD276 | 1.046049503 | 0.003615025 | Up |
| ENST00000560958 | RP11-752G15.9 | 1.055714007 | 0.003315327 | Up |
| ENST00000560810 | WHAMMP3 | 1.003075509 | 0.002419427 | Up |
| ENST00000560634 | CSPG4P11 | -1.32830451 | 0.003235045 | Down |
| ENST00000560444 | PIF1 | 1.388521306 | 0.003119383 | Up |
| ENST00000560141 | TRIM69 | 1.703983434 | 0.000336448 | Up |
| ENST00000559514 | CINP | 1.237936381 | 3.98149E-05 | Up |
| ENST00000559037 | CASC4 | 1.570011022 | 0.000105056 | Up |
| ENST00000558881 | EXD1 | 1.238270821 | 0.000258132 | Up |
| ENST00000558383 | DUOX2 | 1.531882056 | 7.67668E-12 | Up |
| ENST00000558289 | MYEF2 | 1.236016465 | 0.000269571 | Up |
| ENST00000557178 | ERO1L | 1.718177445 | 0.000712458 | Up |
| ENST00000557159 | APEX1 | -1.956094593 | 0.003058682 | Down |
| ENST00000555232 | ARHGEF40 | -2.536682855 | 9.75136E-06 | Down |
| ENST00000554677 | MTHFD1 | 1.532376344 | 0.003922652 | Up |
| ENST00000554611 | CYP46A1 | 1.155273338 | 0.001989092 | Up |
| ENST00000554514 | ARHGEF40 | -1.82967889 | 0.000336883 | Down |
| ENST00000553448 | COQ6 | 1.0364147 | 0.00286348 | Up |
| ENST00000551414 | HEATR5A | 1.156062131 | 3.01176E-05 | Up |
| ENST00000550756 | OLA1P3 | 1.09458739 | 0.0018737 | Up |
| ENST00000550473 | DLST | 1.86366869 | 2.83408E-05 | Up |
| ENST00000550121 | SUOX | 1.492474058 | 0.001370522 | Up |
| ENST00000550065 | SUOX | 1.502363041 | 0.000222376 | Up |
| ENST00000548445 | RND1 | 1.174365791 | 0.00372092 | Up |
| ENST00000548283 | TMEM116 | 1.342045815 | 0.002945045 | Up |
| ENST00000547865 | SPATS2 | 1.460639087 | 0.000873716 | Up |
| ENST00000547255 | WDR83 | -1.221842928 | 0.001201561 | Down |
| ENST00000545509 | ITFG2 | 1.968601301 | 0.000106972 | Up |
| ENST00000545087 | AL590822.1 | 1.038243907 | 0.002135708 | Up |
| ENST00000544665 | ITGAM | 1.66269071 | 0.001866577 | Up |
| ENST00000544269 | PLA2G16 | 1.607470337 | 0.004100267 | Up |
| ENST00000544098 | TMEM132A | -1.49115259 | 1.34366E-05 | Down |
| ENST00000543947 | DNAJB13 | -1.633295073 | 2.25174E-05 | Down |
| ENST00000543897 | ZCCHC8 | 1.277152717 | 0.002151025 | Up |
| ENST00000543600 | PTCHD4 | 1.253403502 | 1.11969E-06 | Up |
| ENST00000542459 | PBXIP1 | 1.760183792 | 0.002272509 | Up |
| ENST00000541475 | RBM7 | 1.093975114 | 9.09196E-05 | Up |
| ENST00000540876 | CLDN2 | -1.398866252 | 0.003658715 | Down |
| ENST00000540112 | TMEM132A | -1.075034461 | 0.001907116 | Down |
| ENST00000539570 | FBXL22 | -1.759996012 | 7.95897E-07 | Down |
| ENST00000538714 | PLEKHA5 | 1.137841801 | 0.000564819 | Up |
| ENST00000538629 | SCAMP1 | 1.806556854 | 7.57162E-05 | Up |
| ENST00000538120 | CLIP1 | 1.153913623 | 0.001026264 | Up |
| ENST00000537694 | PPP1CA | 1.890670116 | 2.25782E-06 | Up |
| ENST00000536545 | HMGA2 | 1.123129034 | 8.50971E-05 | Up |
| ENST00000535529 | MRPL48 | 1.182569235 | 0.001128335 | Up |
| ENST00000535274 | B3GNT4 | -1.439219397 | 9.84704E-07 | Down |
| ENST00000535262 | SEZ6 | -1.053761903 | 0.000947057 | Down |
| ENST00000535052 | CCDC77 | 1.571030135 | 0.001439086 | Up |
| ENST00000533453 | DCBLD1 | 1.630935628 | 0.000154259 | Up |
| ENST00000532860 | HINFP | 1.262660961 | 0.000523989 | Up |
| ENST00000532485 | ACER3 | 1.015393102 | 0.00180029 | Up |
| ENST00000532312 | HINFP | 1.438916075 | 0.001339211 | Up |
| ENST00000531649 | C1orf106 | 1.073888319 | 4.47032E-07 | Up |
| ENST00000531346 | TMEM136 | 1.21988128 | 0.001747586 | Up |
| ENST00000530529 | KDELC2 | 1.132027045 | 0.000148165 | Up |
| ENST00000528823 | PHLDB1 | 1.133478327 | 0.00393832 | Up |
| ENST00000527676 | ETS1 | 1.433879326 | 0.004035368 | Up |
| ENST00000527278 | ZNF273 | 1.300395556 | 4.88615E-08 | Up |
| ENST00000526990 | EHBP1L1 | -2.392459083 | 0.000634164 | Down |
| ENST00000526794 | DDX10 | 1.082244057 | 0.000032597 | Up |
| ENST00000526393 | FAM86C1 | -1.113033899 | 0.000618336 | Down |
| ENST00000526353 | SBF2 | 1.115809936 | 0.000290612 | Up |
| ENST00000525697 | SBF2 | -1.181280566 | 0.000953542 | Down |
| ENST00000525593 | NADSYN1 | 1.574627376 | 2.63296E-05 | Up |
| ENST00000525443 | LTBP3 | 1.78446135 | 6.88927E-06 | Up |
| ENST00000525427 | PHLDB1 | 1.264964228 | 0.00149474 | Up |
| ENST00000524967 | NME7 | 1.067134886 | 0.001422136 | Up |
| ENST00000524750 | CD82 | 1.259482995 | 2.10069E-05 | Up |
| ENST00000524666 | DPF2 | 1.544942756 | 0.0008395 | Up |
| ENST00000524391 | KCNV1 | 1.290053723 | 0.00052086 | Up |
| ENST00000522362 | PNMA2 | 1.151805206 | 5.74437E-06 | Up |
| ENST00000521580 | SMIM12 | 1.327218587 | 0.000512745 | Up |
| ENST00000520915 | RP11-347C12.1 | -3.082647379 | 0.000188371 | Down |
| ENST00000520704 | HGSNAT | 1.556673076 | 0.001873315 | Up |
| ENST00000518164 | RPL30 | 1.685093441 | 0.002447252 | Up |
| ENST00000517381 | STK10 | 1.409971499 | 0.000191438 | Up |
| ENST00000517360 | STK10 | 1.671590467 | 0.000430355 | Up |
| ENST00000515646 | TADA2B | -1.319010912 | 0.00152935 | Down |
| ENST00000515535 | ZNF135 | -1.331781793 | 2.29422E-05 | Down |
| ENST00000514179 | RAPGEF6 | 1.376551958 | 0.003316827 | Up |
| ENST00000513654 | EXOSC9 | 1.859783923 | 0.00032711 | Up |
| ENST00000513291 | ATR | 1.362419117 | 7.72659E-08 | Up |
| ENST00000513146 | TRIM52 | 1.052978962 | 0.000489344 | Up |
| ENST00000512675 | RP11-826N14.2 | -1.358316664 | 0.003664179 | Down |
| ENST00000512507 | H2AFY | 1.865854588 | 0.003004312 | Up |
| ENST00000510834 | BRIX1 | -1.297112994 | 1.40028E-06 | Down |
| ENST00000510711 | RICTOR | 1.387473501 | 0.003936124 | Up |
| ENST00000510450 | ROPN1B | -1.425509119 | 0.003223811 | Down |
| ENST00000509137 | FBXO27 | 1.296933004 | 0.000535683 | Up |
| ENST00000508278 | MRPL48 | 1.224028177 | 0.000732284 | Up |
| ENST00000507754 | NDUFA13 | -1.802819864 | 0.001992373 | Down |
| ENST00000506487 | MRPL3 | 1.269265286 | 0.00017806 | Up |
| ENST00000506437 | ITGA3 | 1.501797457 | 0.000257301 | Up |
| ENST00000506387 | KCNG1 | 1.298672674 | 0.002900271 | Up |
| ENST00000505412 | ZCCHC4 | 1.505751503 | 0.0013401 | Up |
| ENST00000504763 | ARSK | 1.250539942 | 0.00054758 | Up |
| ENST00000504157 | POLR2J3 | 1.035280108 | 0.000368955 | Up |
| ENST00000503874 | LINC00923 | 1.321922477 | 9.17413E-05 | Up |
| ENST00000503859 | RPS6KA2 | -1.988163049 | 3.95028E-06 | Down |
| ENST00000503498 | EFCAB12 | 1.203176166 | 0.000487684 | Up |
| ENST00000502891 | C12orf43 | 1.040889267 | 0.0029438 | Up |
| ENST00000502805 | C4orf21 | 1.054810007 | 0.003576492 | Up |
| ENST00000501515 | RIMS2 | 1.340136424 | 0.000779626 | Up |
| ENST00000500728 | LRPAP1 | 1.102768798 | 0.003149455 | Up |
| ENST00000497070 | FAM131A | 1.118661873 | 0.001333326 | Up |
| ENST00000496912 | TES | 1.957477815 | 0.000876795 | Up |
| ENST00000496878 | MTERFD2 | -1.597816279 | 0.00083483 | Down |
| ENST00000496656 | LHFPL5 | 1.117271825 | 4.02376E-05 | Up |
| ENST00000494927 | RASGRP3 | 1.301936926 | 0.002116593 | Up |
| ENST00000494079 | FAM133B | 1.130877163 | 0.003906859 | Up |
| ENST00000493657 | PUSL1 | -1.367137004 | 0.002861133 | Down |
| ENST00000493423 | SLC37A3 | 1.013490191 | 0.001876058 | Up |
| ENST00000492956 | LEPRE1 | 1.509802107 | 0.001489738 | Up |
| ENST00000492915 | NLRP12 | 1.47726898 | 0.000247202 | Up |
| ENST00000491265 | MZT2A | 1.303924959 | 0.001540078 | Up |
| ENST00000489786 | AQP6 | 1.344629638 | 0.002805308 | Up |
| ENST00000488519 | NAIF1 | 1.335598496 | 3.76125E-08 | Up |
| ENST00000486727 | EXOSC7 | 1.123949631 | 2.38559E-05 | Up |
| ENST00000486212 | MPP4 | 2.003116398 | 1.84808E-08 | Up |
| ENST00000486028 | HMG20B | 1.75592737 | 0.000442774 | Up |
| ENST00000485714 | POMGNT1 | 1.59048035 | 2.72306E-13 | Up |
| ENST00000485488 | EVA1C | -1.669530146 | 0.002385986 | Down |
| ENST00000484301 | MKNK1 | 1.187385204 | 0.001721706 | Up |
| ENST00000483782 | HFE | 2.106519936 | 0.000395991 | Up |
| ENST00000483477 | ARMC9 | 1.216592132 | 1.36598E-05 | Up |
| ENST00000482704 | GPAT2 | -1.158603165 | 2.91149E-05 | Down |
| ENST00000482533 | RCAN1 | 1.75315773 | 0.001918657 | Up |
| ENST00000482280 | MSH5 | 1.524535004 | 0.002360462 | Up |
| ENST00000481865 | TNK2 | 1.330099523 | 0.003947283 | Up |
| ENST00000481410 | C7orf49 | 1.072375551 | 0.001661869 | Up |
| ENST00000480982 | DMTF1 | -1.192718228 | 2.18501E-05 | Down |
| ENST00000480395 | TRIM22 | 1.366067243 | 0.00049729 | Up |
| ENST00000480186 | CA6 | -1.339452269 | 0.002965419 | Down |
| ENST00000480170 | LIPT1 | 1.162247736 | 0.001596023 | Up |
| ENST00000479988 | CPS1 | 1.005312465 | 0.000396617 | Up |
| ENST00000479978 | L3MBTL2 | 1.008305771 | 0.002747486 | Up |
| ENST00000479183 | ECHDC2 | 1.35960975 | 1.51054E-09 | Up |
| ENST00000478503 | SH3D19 | -1.109905718 | 0.000417762 | Down |
| ENST00000476618 | MLTK | 1.13969552 | 0.001833956 | Up |
| ENST00000476243 | CENPV | -1.004458911 | 1.09838E-05 | Down |
| ENST00000475969 | DFFB | 1.18966858 | 0.000170672 | Up |
| ENST00000475941 | DGCR8 | -1.222660142 | 3.9054E-07 | Down |
| ENST00000475922 | LPIN1 | 1.049177593 | 0.000335328 | Up |
| ENST00000475687 | SLC12A9 | 1.289848398 | 1.21757E-05 | Up |
| ENST00000475185 | TMEM241 | 1.086154653 | 0.003637047 | Up |
| ENST00000475031 | C7orf63 | 1.443268887 | 0.001347816 | Up |
| ENST00000474657 | FKBP1A | -2.352812777 | 0.002416904 | Down |
| ENST00000474447 | MCOLN3 | 1.471939056 | 0.001010264 | Up |
| ENST00000474136 | APP | 2.620450783 | 0.001180425 | Up |
| ENST00000473507 | PSD | -1.652291394 | 0.000640861 | Down |
| ENST00000472953 | BAZ2B | 1.004653714 | 0.000912109 | Up |
| ENST00000472855 | NAPB | 1.44078084 | 0.000796058 | Up |
| ENST00000471668 | MYT1L | 1.212902768 | 0.000017124 | Up |
| ENST00000471236 | PTPLB | 1.930102105 | 4.78495E-06 | Up |
| ENST00000471144 | DDX27 | 1.05018101 | 0.000025372 | Up |
| ENST00000470730 | YIPF6 | 1.051501563 | 5.13034E-09 | Up |
| ENST00000470703 | PRR3 | 2.304705018 | 0.000225456 | Up |
| ENST00000470068 | CCDC144A | 1.394346734 | 0.001030604 | Up |
| ENST00000469702 | SLC35F5 | 1.043226009 | 0.003722319 | Up |
| ENST00000469543 | VIM | -1.462954512 | 0.002437946 | Down |
| ENST00000469491 | FNDC3B | 1.154908391 | 0.002553472 | Up |
| ENST00000468468 | TSPAN4 | -1.189639478 | 0.002627135 | Down |
| ENST00000466992 | AK9 | 1.062971401 | 0.000247098 | Up |
| ENST00000465950 | SCAF11 | -1.645852423 | 0.001694785 | Down |
| ENST00000465445 | AP1G2 | -1.044453735 | 0.00235735 | Down |
| ENST00000464185 | SUV420H2 | 1.261888911 | 0.003248501 | Up |
| ENST00000463909 | LZTR1 | -1.35414623 | 5.36098E-08 | Down |
| ENST00000463718 | SLCO1A2 | 1.767171634 | 0.000409609 | Up |
| ENST00000463586 | PRRC2C | 1.1547172 | 7.68337E-06 | Up |
| ENST00000463436 | SFI1 | 1.03501256 | 0.000211326 | Up |
| ENST00000463428 | PHACTR4 | 1.889591001 | 0.001224232 | Up |
| ENST00000463137 | RP11-108K14.4 | 1.063857495 | 1.22822E-06 | Up |
| ENST00000462616 | PMPCA | 1.224047578 | 0.000109151 | Up |
| ENST00000462332 | GTPBP1 | -1.313111248 | 0.002147467 | Down |
| ENST00000461790 | RCSD1 | 1.510268789 | 0.000626369 | Up |
| ENST00000460933 | RYK | 1.357311304 | 0.000301833 | Up |
| ENST00000460508 | LBX2 | -1.38858623 | 0.002958751 | Down |
| ENST00000460362 | ATP5C1 | 1.976625005 | 0.000574056 | Up |
| ENST00000459961 | FHAD1 | -2.41868483 | 0.000311792 | Down |
| ENST00000459901 | ANKMY1 | 1.07503501 | 9.88526E-05 | Up |
| ENST00000458198 | CDKN2AIPNL | -1.46600506 | 5.67552E-07 | Down |
| ENST00000456951 | TSGA13 | 1.141281035 | 0.002111727 | Up |
| ENST00000456932 | DNM1P51 | -1.228752356 | 0.002310076 | Down |
| ENST00000456337 | AC009892.10 | 1.059939939 | 0.003417311 | Up |
| ENST00000455764 | FAM27E3 | -1.949418518 | 0.000230725 | Down |
| ENST00000455730 | LDHBP1 | 1.449314588 | 0.000925829 | Up |
| ENST00000453355 | ZNF385C | 1.934425157 | 4.37745E-05 | Up |
| ENST00000449142 | SLC24A1 | 2.614590144 | 0.001874825 | Up |
| ENST00000448299 | ZNF669 | 1.331772565 | 0.001084391 | Up |
| ENST00000447648 | TECPR1 | 1.458406808 | 0.001320072 | Up |
| ENST00000447279 | ZKSCAN7 | 1.909184413 | 2.12234E-05 | Up |
| ENST00000445820 | CR759762.1 | 1.799542293 | 0.002862782 | Up |
| ENST00000445217 | ZNF793 | 1.077462382 | 0.000652073 | Up |
| ENST00000443002 | POLR2F | 1.594661238 | 0.001417084 | Up |
| ENST00000439871 | CXCR2P1 | 1.675725135 | 0.000493 | Up |
| ENST00000439642 | KIAA0226L | -3.703313961 | 1.07301E-06 | Down |
| ENST00000438197 | SYTL2 | 1.264266375 | 2.00802E-08 | Up |
| ENST00000437104 | ABCC10 | 1.011332707 | 0.000808382 | Up |
| ENST00000436928 | SENP6 | 1.251313646 | 0.000996085 | Up |
| ENST00000436693 | TLR6 | 1.004953132 | 0.00398608 | Up |
| ENST00000436580 | TRIM64EP | 1.510532672 | 1.96613E-05 | Up |
| ENST00000436367 | ATXN1 | 1.291485574 | 0.004037685 | Up |
| ENST00000436115 | NDUFS7 | 1.741949008 | 6.76783E-06 | Up |
| ENST00000433891 | MLLT6 | 1.060918591 | 0.000366867 | Up |
| ENST00000433570 | SLC16A1 | 2.000735137 | 0.001481971 | Up |
| ENST00000430711 | COL28A1 | 1.173205822 | 6.86157E-06 | Up |
| ENST00000429591 | ZNF713 | 1.197873112 | 3.37435E-09 | Up |
| ENST00000427638 | MED15P9 | 1.425269175 | 0.001512433 | Up |
| ENST00000427525 | SCAND2P | -1.572960555 | 0.003368483 | Down |
| ENST00000424347 | CEP78 | 1.643475798 | 0.003916716 | Up |
| ENST00000422953 | RP11-39K24.9 | 1.064712315 | 0.002370297 | Up |
| ENST00000421699 | GOLGA2 | 1.097261179 | 0.001210164 | Up |
| ENST00000421177 | NSG1 | 1.139646513 | 0.000366948 | Up |
| ENST00000418681 | NUBPL | 1.172306556 | 0.002741811 | Up |
| ENST00000417536 | SH3BP1 | 1.099903443 | 0.000212689 | Up |
| ENST00000415933 | PPIA | 2.680462069 | 0.000835951 | Up |
| ENST00000415452 | KIAA0226 | 1.697299248 | 0.001037234 | Up |
| ENST00000415430 | VSIG1 | 2.634810167 | 0.001723788 | Up |
| ENST00000415349 | KRT18P39 | -1.876006397 | 4.72445E-05 | Down |
| ENST00000415191 | C2CD3 | 1.84091287 | 9.47923E-05 | Up |
| ENST00000414696 | C6orf141 | 1.355068653 | 0.000086202 | Up |
| ENST00000412782 | RP11-307I14.3 | -1.015064203 | 0.002579251 | Down |
| ENST00000411958 | TMEM120B | 3.284189372 | 3.98204E-06 | Up |
| ENST00000409734 | C2orf68 | 1.061106339 | 1.50118E-06 | Up |
| ENST00000409458 | GPNMB | 1.661295798 | 0.000321161 | Up |
| ENST00000409239 | LY6G6E | 1.789886984 | 0.00013101 | Up |
| ENST00000400773 | FBLIM1 | 1.128982155 | 0.000220953 | Up |
| ENST00000399799 | ROCK1 | 1.222870432 | 0.000280954 | Up |
| ENST00000399540 | AC027763.2 | 2.239607608 | 1.29898E-05 | Up |
| ENST00000397762 | RAB2B | 1.391113414 | 0.003462188 | Up |
| ENST00000397061 | NUDT19 | 1.025173762 | 0.000646266 | Up |
| ENST00000396556 | OSBPL10 | 1.079054829 | 0.000098181 | Up |
| ENST00000395863 | BMP7 | 1.209373314 | 0.000778549 | Up |
| ENST00000395699 | PURB | 1.156546719 | 0.001020212 | Up |
| ENST00000395468 | MAPK1IP1L | 1.280102631 | 4.66623E-05 | Up |
| ENST00000394684 | SGMS2 | 1.953479031 | 0.003432396 | Up |
| ENST00000393518 | FAM153A | 1.625296667 | 0.001027981 | Up |
| ENST00000393067 | POU2AF1 | 1.526406723 | 3.43776E-07 | Up |
| ENST00000393056 | DBH | -1.301436425 | 0.001077285 | Down |
| ENST00000392514 | RPLP0 | -2.631781672 | 0.001096416 | Down |
| ENST00000392495 | SPPL3 | 1.253242932 | 0.001162126 | Up |
| ENST00000383811 | CRELD1 | -1.359374152 | 0.000638238 | Down |
| ENST00000383694 | FILIP1L | -1.030705464 | 0.000144829 | Down |
| ENST00000381800 | LOH12CR2 | 1.170807618 | 0.002500317 | Up |
| ENST00000380314 | RXFP2 | 1.364052004 | 0.000530665 | Up |
| ENST00000380265 | HERC6 | 1.926713955 | 0.000127782 | Up |
| ENST00000380152 | BRCA2 | 1.583454233 | 0.00059554 | Up |
| ENST00000379882 | DDX58 | -1.863326262 | 0.00011794 | Down |
| ENST00000379121 | BCAP29 | 1.251223759 | 0.003462444 | Up |
| ENST00000378719 | SEPT8 | -1.470230327 | 0.000161903 | Down |
| ENST00000378268 | EBPL | 1.036783162 | 0.000773257 | Up |
| ENST00000378244 | DEPDC4 | 1.072855042 | 8.98387E-05 | Up |
| ENST00000378230 | CEP104 | 1.138630317 | 0.003281752 | Up |
| ENST00000378192 | TLN1 | 1.867937647 | 6.73669E-05 | Up |
| ENST00000378165 | NMT2 | 1.435178657 | 1.64332E-05 | Up |
| ENST00000377957 | FBXO48 | 1.013608247 | 0.000245985 | Up |
| ENST00000377424 | SLC2A5 | 1.137556198 | 0.001155858 | Up |
| ENST00000377411 | GPR157 | 1.250864772 | 9.55417E-05 | Up |
| ENST00000377038 | DFFA | 1.030853112 | 0.003993667 | Up |
| ENST00000375960 | ZCCHC6 | 1.88334957 | 0.002274373 | Up |
| ENST00000375258 | METTL8 | 1.089444601 | 5.91457E-05 | Up |
| ENST00000374296 | PAQR7 | 1.584643192 | 1.30181E-07 | Up |
| ENST00000374278 | SLC30A2 | 1.28219421 | 0.002231964 | Up |
| ENST00000374212 | SLC31A1 | 1.009423293 | 0.000150799 | Up |
| ENST00000374199 | PRPF4 | 1.447105269 | 0.000756136 | Up |
| ENST00000373186 | MOCS1 | 2.968418145 | 0.000362974 | Up |
| ENST00000373014 | UTP11L | 1.69338388 | 4.6098E-06 | Up |
| ENST00000372977 | TAF8 | 1.107037114 | 0.000165692 | Up |
| ENST00000372405 | PTPRF | 1.143623002 | 4.71554E-06 | Up |
| ENST00000371752 | ZNFX1 | -1.139482287 | 0.00069551 | Down |
| ENST00000371655 | RAB3B | 1.220580564 | 0.000382472 | Up |
| ENST00000371602 | ADNP | -1.356486764 | 5.48586E-05 | Down |
| ENST00000371335 | LAMP2 | 1.380759201 | 0.003411358 | Up |
| ENST00000371265 | BSND | 2.359124013 | 0.000438695 | Up |
| ENST00000371177 | TM2D1 | 1.692451224 | 0.001674834 | Up |
| ENST00000370692 | LAMA5 | 1.257138163 | 0.002765227 | Up |
| ENST00000370631 | PI4K2A | -1.114977057 | 0.000275528 | Down |
| ENST00000369880 | AS3MT | 1.185347222 | 1.89374E-05 | Up |
| ENST00000369864 | AMIGO1 | -1.210051546 | 1.68302E-06 | Down |
| ENST00000369769 | KCNA3 | -1.736540801 | 8.63559E-12 | Down |
| ENST00000369603 | SMNDC1 | -1.259898256 | 6.7013E-14 | Down |
| ENST00000368847 | KIAA1919 | 1.194211948 | 0.001048626 | Up |
| ENST00000368026 | F11R | 1.412522826 | 1.2988E-06 | Up |
| ENST00000367812 | MYB | 1.719661542 | 0.000106434 | Up |
| ENST00000367419 | GINM1 | 1.141851391 | 0.000930924 | Up |
| ENST00000367160 | DSTYK | 1.146977902 | 0.000949269 | Up |
| ENST00000366997 | LPGAT1 | 1.610657032 | 0.000907236 | Up |
| ENST00000366932 | RRP15 | 1.076098609 | 0.000283775 | Up |
| ENST00000366903 | HLX | 1.619786615 | 6.26117E-05 | Up |
| ENST00000366847 | FGFR1OP | 1.11303048 | 0.00260454 | Up |
| ENST00000366788 | C1orf95 | -1.506962046 | 0.001368434 | Down |
| ENST00000366606 | RBM34 | 1.443503639 | 4.05397E-10 | Up |
| ENST00000366304 | HMGB1P39 | 1.114263849 | 0.003438396 | Up |
| ENST00000366286 | YBX3 | 1.340780382 | 0.000712306 | Up |
| ENST00000366277 | TMEM180 | 1.344131797 | 8.92242E-05 | Up |
| ENST00000366221 | AL645728.1 | 1.44024518 | 0.000126229 | Up |
| ENST00000362079 | MT-CO3 | 1.235181784 | 0.000400281 | Up |
| ENST00000361851 | MT-ATP8 | 1.800333162 | 0.001239205 | Up |
| ENST00000361789 | MT-CYB | 1.49082665 | 0.000111203 | Up |
| ENST00000361677 | RUNDC1 | 1.042034089 | 0.00275202 | Up |
| ENST00000361627 | ARHGAP11A | 1.591975144 | 0.000783459 | Up |
| ENST00000361354 | NCKAP1 | 1.541439024 | 0.000684526 | Up |
| ENST00000360943 | UBE2Z | -1.420671377 | 0.001383641 | Down |
| ENST00000360737 | FLJ27365 | 1.226272442 | 1.39208E-05 | Up |
| ENST00000360506 | ANGEL2 | 1.16347051 | 0.00087574 | Up |
| ENST00000360428 | DSC3 | 1.431204534 | 1.89319E-06 | Up |
| ENST00000359927 | FRMPD1 | 1.062883895 | 3.45263E-05 | Up |
| ENST00000359866 | DDA1 | -1.871345984 | 0.0004366 | Down |
| ENST00000359543 | EMP2 | 1.050547 | 0.003118728 | Up |
| ENST00000359236 | VSIG10 | 1.642259976 | 0.00056448 | Up |
| ENST00000358869 | FAM114A1 | 1.287412868 | 0.00318373 | Up |
| ENST00000357869 | ADCY6 | 1.935329054 | 9.06968E-05 | Up |
| ENST00000357618 | HFE | 1.436215042 | 0.000136891 | Up |
| ENST00000357096 | ACSL6 | 1.505942257 | 0.002199129 | Up |
| ENST00000356951 | PRDX3 | -1.204374278 | 0.000356153 | Down |
| ENST00000356189 | ACBD7 | 1.380108303 | 0.000748928 | Up |
| ENST00000356178 | ZNF527 | 1.483165416 | 0.000191452 | Up |
| ENST00000355797 | MXRA7 | 1.094440563 | 0.000103714 | Up |
| ENST00000355028 | BCL2L13 | -1.547893221 | 0.000235859 | Down |
| ENST00000354485 | KANK1 | 1.394724739 | 0.002557764 | Up |
| ENST00000354479 | C9orf131 | -1.173202473 | 0.003410623 | Down |
| ENST00000354280 | EPPIN | 1.142494554 | 0.000682583 | Up |
| ENST00000354278 | NLRP12 | 1.243634619 | 0.00027116 | Up |
| ENST00000353411 | SKP1 | -1.213303269 | 0.000938942 | Down |
| ENST00000350777 | HINFP | 1.245077761 | 0.000940938 | Up |
| ENST00000347970 | TM2D3 | -2.010140486 | 0.001588618 | Down |
| ENST00000344721 | NR3C2 | 1.481918396 | 0.00311781 | Up |
| ENST00000344135 | TRIO | 2.496328574 | 0.000675452 | Up |
| ENST00000341513 | NUGGC | 1.447429772 | 0.002838806 | Up |
| ENST00000340625 | CXorf23 | 1.394008861 | 4.90999E-06 | Up |
| ENST00000340415 | RAB37 | -1.935623473 | 0.000246705 | Down |
| ENST00000339834 | C10orf32 | 1.122800591 | 0.00067345 | Up |
| ENST00000339569 | MSL1 | -1.232329763 | 7.76665E-16 | Down |
| ENST00000337514 | IGF1 | 2.220669583 | 0.001091101 | Up |
| ENST00000334815 | HPDL | -1.460409457 | 6.37103E-05 | Down |
| ENST00000334801 | BCL9L | -1.305599231 | 0.000382982 | Down |
| ENST00000334528 | FMN1 | 1.051303228 | 0.000610882 | Up |
| ENST00000334241 | ZNF555 | 1.566610738 | 0.00014389 | Up |
| ENST00000330689 | WDR5B | -1.195315909 | 3.35692E-08 | Down |
| ENST00000330342 | ATP6V0A2 | 1.353244931 | 1.49336E-08 | Up |
| ENST00000330276 | SV2B | 1.367605624 | 5.68195E-05 | Up |
| ENST00000328933 | SYNGR1 | -1.055004611 | 2.29765E-06 | Down |
| ENST00000327590 | FOXP1 | 1.612122839 | 9.42595E-06 | Up |
| ENST00000327381 | XKR4 | 1.404270149 | 0.000230389 | Up |
| ENST00000326877 | LCORL | 2.074262274 | 5.89791E-06 | Up |
| ENST00000325233 | C8orf34 | 1.407628826 | 0.000029477 | Up |
| ENST00000324198 | ADRBK2 | 1.059807152 | 0.001970953 | Up |
| ENST00000321844 | RP11-111M22.2 | 1.007007468 | 0.001403535 | Up |
| ENST00000319637 | EPHA10 | 1.182255004 | 2.9859E-07 | Up |
| ENST00000318636 | CA5B | 1.051568574 | 7.52141E-06 | Up |
| ENST00000318312 | BBS1 | 1.70330632 | 0.003896201 | Up |
| ENST00000317449 | LRRN4CL | 1.099886302 | 0.00051748 | Up |
| ENST00000317310 | CD300LG | 1.33824231 | 0.000105614 | Up |
| ENST00000316334 | LNX2 | 1.441595251 | 9.30699E-05 | Up |
| ENST00000314675 | UBXN11 | 1.404135458 | 1.76695E-17 | Up |
| ENST00000311630 | FBXO45 | 1.070858759 | 0.00026914 | Up |
| ENST00000311623 | C4orf26 | 1.501669316 | 0.000819774 | Up |
| ENST00000311620 | ANKS4B | 1.704712551 | 0.000454666 | Up |
| ENST00000310160 | FUT1 | 2.234364822 | 1.49189E-07 | Up |
| ENST00000309951 | ACO1 | 1.537712558 | 0.001329953 | Up |
| ENST00000309934 | SEC22A | 1.310907209 | 0.002472731 | Up |
| ENST00000307602 | HOOK3 | 1.172739717 | 0.001631695 | Up |
| ENST00000306480 | TMEM192 | 1.389800147 | 0.000748916 | Up |
| ENST00000306077 | TMEM43 | -1.414682346 | 9.45633E-06 | Down |
| ENST00000305368 | EPB41L4A | 1.708385695 | 9.86225E-05 | Up |
| ENST00000304058 | DTWD2 | 1.447394213 | 0.001688095 | Up |
| ENST00000302495 | HTRA4 | 2.106821988 | 0.000844798 | Up |
| ENST00000301765 | VPS37C | -2.27291955 | 2.58122E-09 | Down |
| ENST00000301357 | CD209 | 1.120532898 | 0.001367797 | Up |
| ENST00000299853 | POLR3E | -1.5030117 | 0.000466547 | Down |
| ENST00000299381 | ANAPC16 | 1.405193234 | 0.000729738 | Up |
| ENST00000299138 | VPS35 | 2.182671998 | 0.000450196 | Up |
| ENST00000299084 | SPRED1 | 1.175859628 | 0.002142148 | Up |
| ENST00000298310 | NEMF | -1.07506399 | 1.81773E-07 | Down |
| ENST00000298292 | DNAAF2 | 3.190854694 | 0.000124281 | Up |
| ENST00000298198 | PGM2L1 | 1.136181106 | 0.000522578 | Up |
| ENST00000296795 | TLR3 | 1.515642173 | 3.52876E-09 | Up |
| ENST00000296658 | CMBL | 1.144060508 | 8.15004E-05 | Up |
| ENST00000296632 | STARD4 | -1.102502275 | 0.00039325 | Down |
| ENST00000296575 | HHIP | 1.172865821 | 0.002150625 | Up |
| ENST00000296506 | SCRG1 | 1.095554332 | 0.003616718 | Up |
| ENST00000295958 | SMIM14 | 1.053149147 | 0.000533493 | Up |
| ENST00000295628 | LRRC58 | 1.140416919 | 1.02287E-06 | Up |
| ENST00000295465 | ANTXR2 | -1.543534407 | 0.000423489 | Down |
| ENST00000294072 | CYB561A3 | -1.497327286 | 0.003450601 | Down |
| ENST00000288561 | UBN2 | 1.406825959 | 0.000468006 | Up |
| ENST00000281932 | GPATCH11 | 1.232786058 | 0.000916906 | Up |
| ENST00000281508 | CCDC122 | 1.140625777 | 0.002117172 | Up |
| ENST00000281441 | TMEM45B | -1.226776529 | 0.000167356 | Down |
| ENST00000280734 | TMEM86A | -1.165323548 | 0.00154046 | Down |
| ENST00000277942 | NPFFR1 | 1.212495564 | 0.003439523 | Up |
| ENST00000277491 | AL354898.1 | 2.659011112 | 0.000321417 | Up |
| ENST00000273666 | STXBP5L | 2.234532801 | 0.001126113 | Up |
| ENST00000271450 | FCGR2A | 2.182091853 | 0.003947412 | Up |
| ENST00000270617 | ZNF473 | -1.441642571 | 0.001165627 | Down |
| ENST00000270189 | PLAT | -1.075513883 | 0.001947755 | Down |
| ENST00000267202 | VPS37B | -1.08544915 | 0.00022283 | Down |
| ENST00000266085 | TIMP3 | -3.036593313 | 1.08253E-05 | Down |
| ENST00000265641 | CPT1A | 1.154739886 | 1.24863E-05 | Up |
| ENST00000265068 | KIAA1257 | 1.473599107 | 0.000118974 | Up |
| ENST00000261867 | SLC30A4 | -2.475064456 | 0.00045169 | Down |
| ENST00000261491 | DGKH | 1.309993376 | 2.34637E-05 | Up |
| ENST00000261326 | MOCOS | 1.26470002 | 0.004140407 | Up |
| ENST00000261275 | FAM189A1 | -1.264439387 | 0.000297589 | Down |
| ENST00000254658 | PER2 | 1.147836761 | 0.000676932 | Up |
| ENST00000252809 | GDF15 | -1.379559143 | 0.003962893 | Down |
| ENST00000252444 | LDLR | 1.41079973 | 9.55803E-06 | Up |
| ENST00000251527 | ESYT2 | 1.506063035 | 0.001179212 | Up |
| ENST00000251269 | ZNF221 | 1.354488856 | 0.001656658 | Up |
| ENST00000249344 | STRIP2 | 1.043210616 | 0.002222565 | Up |
| ENST00000246104 | SCRT2 | -2.012154088 | 0.001331438 | Down |
| ENST00000244571 | AARS2 | 1.056201633 | 3.25325E-05 | Up |
| ENST00000244336 | CEACAM8 | 1.335512381 | 0.000515011 | Up |
| ENST00000238831 | YIPF4 | 1.263505606 | 0.00070498 | Up |
| ENST00000237937 | ZFAND5 | 1.123154177 | 0.000616084 | Up |
| ENST00000237536 | SOGA1 | 1.213255043 | 1.61384E-06 | Up |
| ENST00000229239 | GAPDH | -1.650954301 | 0.002319652 | Down |
| ENST00000223076 | RP11-44M6.3 | 1.050564835 | 0.002988809 | Up |
| ENST00000223073 | RBM28 | 1.612049248 | 7.82955E-08 | Up |
| ENST00000222462 | WNT16 | -1.171564944 | 0.000502706 | Down |
| ENST00000216099 | APOBEC3D | 1.170911032 | 2.57131E-05 | Up |
| ENST00000216044 | GTPBP1 | -1.058339795 | 0.000419394 | Down |
| ENST00000203664 | OTUB2 | -1.17390286 | 2.45783E-10 | Down |
| ENST00000013222 | INMT | 1.159354774 | 7.11209E-06 | Up |

| **Supplementary Table 6. Most relevant networks of the target gene of circRNAs.** | | | | | | | | |
| --- | --- | --- | --- | --- | --- | --- | --- | --- |
| No | Network name | Processes | Size | Target | Pathways | p‑Value | zScore | gScore |
| 1 | [**WNT**, Vimentin, Beta‑catenin, Tcf(Lef), TCF7L2 (TCF4)](applewebdata://FAE5775C-E8B4-45B3-B33C-5F9BB10BD7E0/cgi/network/net_net.cgi?id=-1683544524) | canonical Wnt signaling pathway (46.8%), regulation of cell proliferation (80.9%), regulation of Wnt signaling pathway (51.1%), regulation of canonical Wnt signaling pathway (46.8%), cell‑cell signaling by wnt (48.9%) | 50 | 2 | 102 | 5.00e‑03 | 5.87 | 133.37 |
| 2 | [RAB2B, FAM131A, SV2B, DOC1, OTUB2](applewebdata://FAE5775C-E8B4-45B3-B33C-5F9BB10BD7E0/cgi/network/net_net.cgi?id=-383063963) | positive regulation of secretion by cell (22.0%), transport (58.5%), initiation of movement involved in cerebral cortex radial glia guided migration (4.9%), cytosolic calcium signaling involved in initiation of cell movement in glial‑mediated radial cell migration (4.9%), sphingolipid mediated signaling pathway (7.3%) | 50 | 26 | 0 | 2.12e‑56 | 79.38 | 79.38 |
| 3 | [ETS1, ZNF273, UNQ1887, ZNF554, COQ6](applewebdata://FAE5775C-E8B4-45B3-B33C-5F9BB10BD7E0/cgi/network/net_net.cgi?id=-915454670) | gene expression (59.6%), cellular component disassembly (23.4%), tRNA transport (10.6%), RNA transport (17.0%), nucleic acid transport (17.0%) | 50 | 21 | 1 | 1.94e‑43 | 64.71 | 65.96 |
